# Microvesicles provide a mechanism for intercellular communication by embryonic stem cells during embryo implantation

**DOI:** 10.1038/ncomms11958

**Published:** 2016-06-15

**Authors:** Laura M. Desrochers, François Bordeleau, Cynthia A. Reinhart-King, Richard A. Cerione, Marc A. Antonyak

**Affiliations:** 1 Department of Molecular Medicine, Cornell University, Ithaca, New York 14853, USA; 2 Meinig School of Biomedical Engineering, Cornell University, Ithaca, New York 14853, USA; 3 Department of Chemistry and Chemical Biology, Cornell University, Ithaca, New York 14853, USA

## Abstract

Communication between the inner cell mass (ICM) and the trophoblast layer of the blastocyst is known to occur, but its functional consequences on early developmental events is unclear. Here we demonstrate that embryonic stem (ES) cells derived from the ICM generate and shed microvesicles (MVs), a major class of extracellular vesicles (EVs), which influence trophoblast behaviour during the implantation process. The MV cargo proteins laminin and fibronectin interact with integrins along the surfaces of the trophoblasts, triggering the activation of two signalling kinases, JNK and FAK, and stimulating trophoblast migration. We further show that injecting MVs isolated from ES cells into blastocysts results in an increase in their implantation efficiency. Thus, these findings highlight a unique mechanism by which ES cells communicate with trophoblasts within the blastocyst to increase their ability to migrate into the uterus, thereby promoting one of the earliest and most important steps during pregnancy.

## Abstract (web summary)

It is unclear how embryonic stem cells (ESC) communicate with surrounding cells during implantation. Here, the authors show that microvesicles (MV) are shed from ESCs, activating integrin and JNK/FAK kinases in trophoblasts, stimulating migration *in vitro*, and injecting MVs enhances blastocyst implantation.

## Graphical abstract

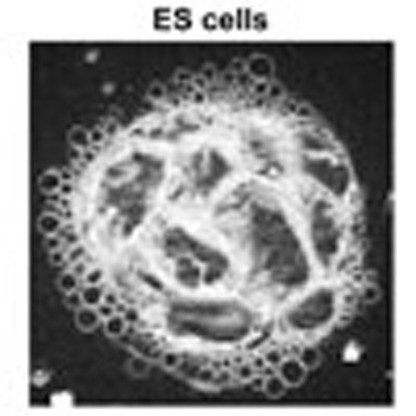

The generation and release (shedding) of extracellular vesicles (EVs) by cells is now appreciated as a major mechanism by which cells communicate with their environment. Many cell types, ranging from embryonic stem (ES) cells^1^,^2^ to highly malignant cancer cells^3^,^4^,^5^, are capable of generating two different classes of EVs, called exosomes and microvesicles (MVs), which can be distinguished by a few physical characteristics as well as the underlying mechanisms responsible for their biogenesis^6^,^7^,^8^. Exosomes range in size from 30–100 nm and are derived from the re-routing of multivesicular bodies destined for degradation in the lysosome to the cell surface where they fuse with the plasma membrane and are released^7^,^9^. MVs, which are also referred to as ectosomes, microparticles, and when produced by cancer cells as tumour-derived MVs or oncosomes, tend to be considerably larger than exosomes (0.2–2 μm in diameter), and are formed and shed directly from the plasma membrane^8^,^10^. EVs have been attracting considerable attention because of the diversity of proteins and nucleic acids that they contain as cargo, including cell surface receptors, cytosolic and nuclear signalling proteins, extracellular matrix proteins, RNA transcripts, microRNAs and even DNA^11^. Moreover, they have the ability to transfer their contents to other cells where they stimulate signalling activities that lead to phenotypic and functional changes in the recipient cells^1^,^3^,^6^,^7^,^12^,^13^,^14^. EVs have been extensively studied in the context of cancer progression, where they have been shown to promote cell growth and survival as well as invasion and metastasis^3^,^8^,^12^,^14^,^15^,^16^,^17^,^18^. However, the importance of EVs in physiological processes is less well understood.

Embryo implantation is a complex process that involves the close communication and interaction between the maternal uterine environment and the blastocyst stage embryo^19^,^20^. A blastocyst is composed of two distinct cell types: the inner cell mass (ICM), which forms the embryo, and the trophectoderm, which surrounds the ICM and eventually forms the placenta^19^. The trophectoderm layer is responsible for initially attaching the blastocyst to the uterine lining, at which point, the trophectoderm, now referred to as trophoblasts, migrates and invades into the uterus to implant the embryo (that is, implantation). The trophoblasts then proliferate extensively and continue to migrate and invade into the uterus to create the placenta, which brings nutrients to the growing embryo^20^. These early developmental events are paramount for the establishment of a successful pregnancy, and errors that occur during implantation can have dire consequences. For example, failure of the trophectoderm to properly implant the embryo often results in spontaneous abortions, whereas improper placental formation has deleterious effects on later stages of pregnancy, potentially causing conditions such as pre-eclampsia and intrauterine growth restriction^21^,^22^.

One of the major aspects of early embryogenesis that has been receiving a good deal of attention concerns to what extent the cells in the ICM of the blastocyst interact with their surroundings to shape fundamental physiological processes underlying normal development^23^,^24^. Here we examine how ES cells, which are derived from the ICM, engage in intercellular communication within the biological context of the blastocyst stage embryo and its implantation into the uterus. We show, using *in vitro* approaches, that ES cells release MVs, which can activate signalling pathways in trophoblasts, leading to enhanced migration. This is accomplished through the interaction of fibronectin and laminin, two extracellular matrix proteins present on the ES cell-derived MVs, with integrins on the surfaces of the trophoblasts. Finally, we show that the injection of ES cell MVs into blastocysts enhances their implantation rates after the embryos are transferred into the uteruses of female mice.

## Results

### ES cells generate and shed MVs

**Figure 1:**
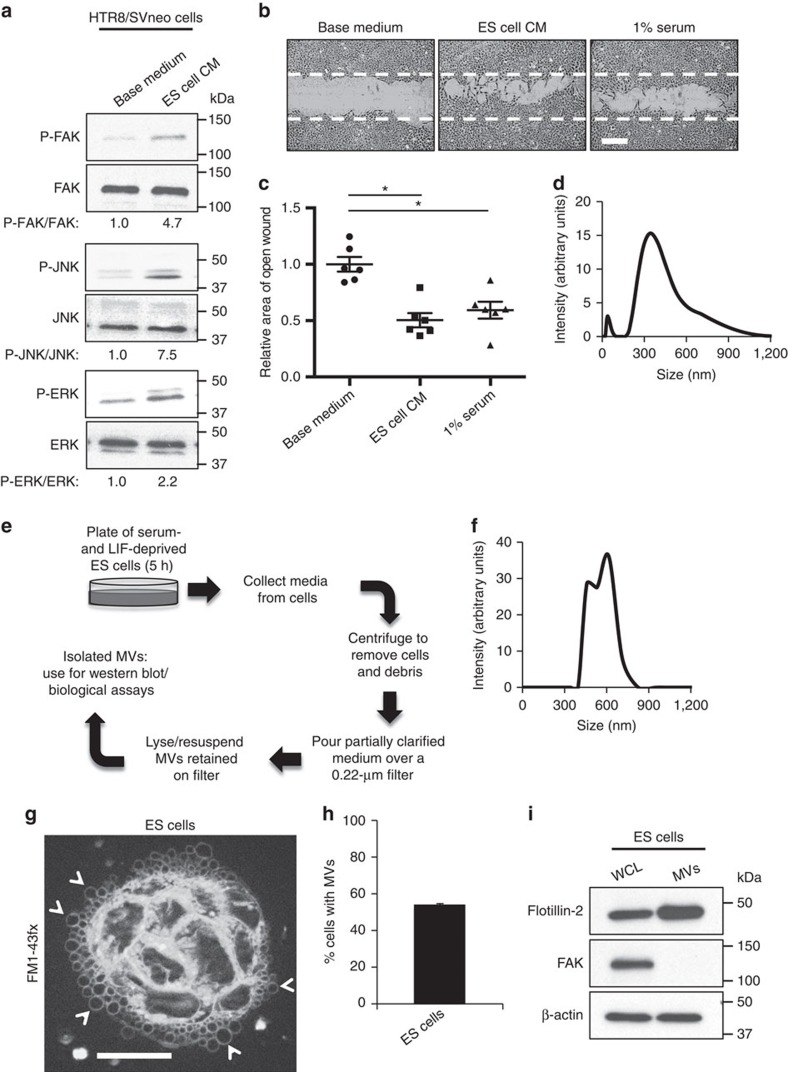
ES cells generate extracellular vesicles. (**a**) Serum-starved HTR8/SVneo trophoblasts treated with either ES cell base medium lacking serum and LIF (Base medium) or with the CM from E14tg2a.4 mouse ES cells cultured in the same medium (ES cell CM) were immunoblotted for phosphorylated FAK (P-FAK), JNK (P-JNK), and ERK (P-ERK). The blots were also probed for total FAK, JNK and ERK. The ratio of each phospho-protein/total protein pair examined was determined and included on the blots. (**b**) Images were taken of wound closure assays performed on HTR8/SVneo cells cultured in ES cell base medium (Base medium), the CM collected from ES cells cultured in the same base medium, or medium containing 1% serum. The dashed line indicates the width of the original wound. Scale bar, 250 μm. (**c**) The assays in (**b**) were quantified and plotted as relative area of open wound. (**d**) Dynamic light scattering plot of the E14tg2a.4 ES cell CM clarified of cells and debris. Note the detection of a ∼30-nm exosome peak and a ∼450-nm MV peak in the CM. (**e**) Procedure for isolating MVs from CM. (**f**) Dynamic light scattering plot of the MV preparation from the E14tg2a.4 ES cell CM. Note the detection of a single peak of ∼600 nm. (**g**) Fluorescence microscopy image of ES cells stained with the membrane dye FM1-43fx. Some of the MVs are denoted with arrowheads. Scale bar, 20 μm (**h**) The percentage of cells in **g** with detectable levels of MVs on their surfaces was determined. (**i**) Lysates of ES cells (whole cell lysate, WCL) and the MVs generated by these cells were immunoblotted for the MV marker flotillin-2, the cytosolic-specific marker FAK, and β-actin as the loading control. All values shown are presented as mean±s.e.m. (*n*≥3 independent experiments for each assay). Differences were analysed using Student's *t*-test; *P<0.05.

An important and as yet unanswered question is whether signalling between the cells that constitute the ICM and the surrounding layer of trophoblasts has a major influence on trophoblast function (that is, migration and/or invasion). As a first step towards addressing this question, we treated the HTR8/SVneo trophoblast cell line^25^ with either ES cell base medium lacking serum and leukaemia inhibitory factor (LIF), or with the same base medium that had been first conditioned by adding it to cultures of the pluripotent, feeder layer-independent E14tg2a.4 ES cell line^26^ (Supplementary Fig. 1a,b) for 5 h, referred to as conditioned medium (CM). It is important to note that the ES cells were thoroughly rinsed with phosphate-buffered saline (PBS) just before being placed in base medium to ensure that the serum and LIF present in the typical ES cell growth medium was removed from the cells and that all components present in the CM were derived from the ES cells. Before being added to recipient cells (for example, trophoblasts), the CM collected was further processed using differential centrifugations to remove cells and cell debris. After treatment with either base medium or CM, the trophoblasts were immunoblotted to detect the phosphorylated (activated) and total forms of signalling proteins frequently implicated in promoting cell migration, including focal adhesion kinase (FAK), c-Jun N-terminal kinase (JNK) and extracellular signal-regulated kinase 1/2 (ERK1/2)^27^,^28^,^29^. Figure 1a shows that the CM (lane labelled ES cell CM) strongly upregulated the phosphorylation levels of FAK (P-FAK) and JNK (P-JNK) compared with cells that were cultured in the ES cell base medium alone (lane labelled Base medium).

Wound closure (migration) assays were then performed to determine whether ES cell CM stimulates trophoblast migration. Wounds were struck through confluent monolayers of HTR8/SVneo trophoblasts placed in ES cell base medium (medium lacking serum and LIF), or in the same medium supplemented with either the CM collected from ES cells or serum. While trophoblasts maintained in only the base medium exhibited minimal migration after 12 h, trophoblasts cultured in CM were capable of migrating into the wound to a degree comparable to that achieved on serum stimulation (Fig. 1b,c). Inhibiting FAK and JNK activation blocked the ability of the ES cell CM to promote trophoblast migration, whereas inhibiting ERK activation had no effect (Supplementary Fig. 2a–f).

Many types of cells are capable of forming and shedding EVs, including MVs and exosomes, into their surroundings. Moreover, we and others have previously shown that MVs and exosomes isolated from CM samples (for example, from cancer cells) are able to initiate a wide variety of changes in recipient cells, including the activation of cellular signalling events that promote cell growth, survival and migration^3^,^12^,^15^,^30^,^31^,^32^. Thus, we considered whether this unique form of cell–cell communication accounted for the ability of the CM from ES cells to affect the signalling activities and functions of trophoblasts.

Following the removal of cells and debris by differential centrifugation, the ES cell CM was analysed by dynamic light scattering (DLS) to determine the sizes of EVs present within these preparations. Figure 1d shows that ES cells produced EVs of two distinct sizes, averaging ∼30 and ∼450 nm. The size of the smaller vesicles corresponds to that of exosomes, while the larger vesicles represent MVs. The CM was then passed through a filter with a 0.22-μm pore size (Fig. 1e). The filter only retained vesicles larger than 0.22 μm in diameter (that is, the MVs), while the smaller exosomes as well as soluble factors were not retained. The MVs were then rinsed extensively with PBS to ensure that any trace amounts of exosomes or soluble proteins remaining were removed from the MV preparation. MVs ranging from ∼350 to 800 nm in diameter were obtained (Fig. 1f). Transmission electron microscopy performed on the isolated MVs showed individual vesicles with sizes that were consistent with the DLS analysis (Supplementary Fig. 3a). Likewise, microscopy experiments using the lipid-binding fluorescent dye FM1-43fx also revealed MVs of varying sizes decorating the surfaces of a majority of the ES cell population (Fig. 1g,h). The MV marker flotillin-2 (Fig. 1i, top panel) was readily detected by immunoblot analysis of these MV preparations, while FAK (middle panel) was only found in the whole-cell lysates (WCL). Moreover, control experiments demonstrated that the MV isolation procedure efficiently removed soluble proteins (for example, growth factors) from our MV preparations (Supplementary Fig. 3b). Collectively, the findings indicate that the ES cell-derived MV preparations were largely homogeneous and devoid of exosomes, cytosolic contamination, as well as freely secreted proteins.

### ES cell MVs enhance trophoblast migration

**Figure 2:**
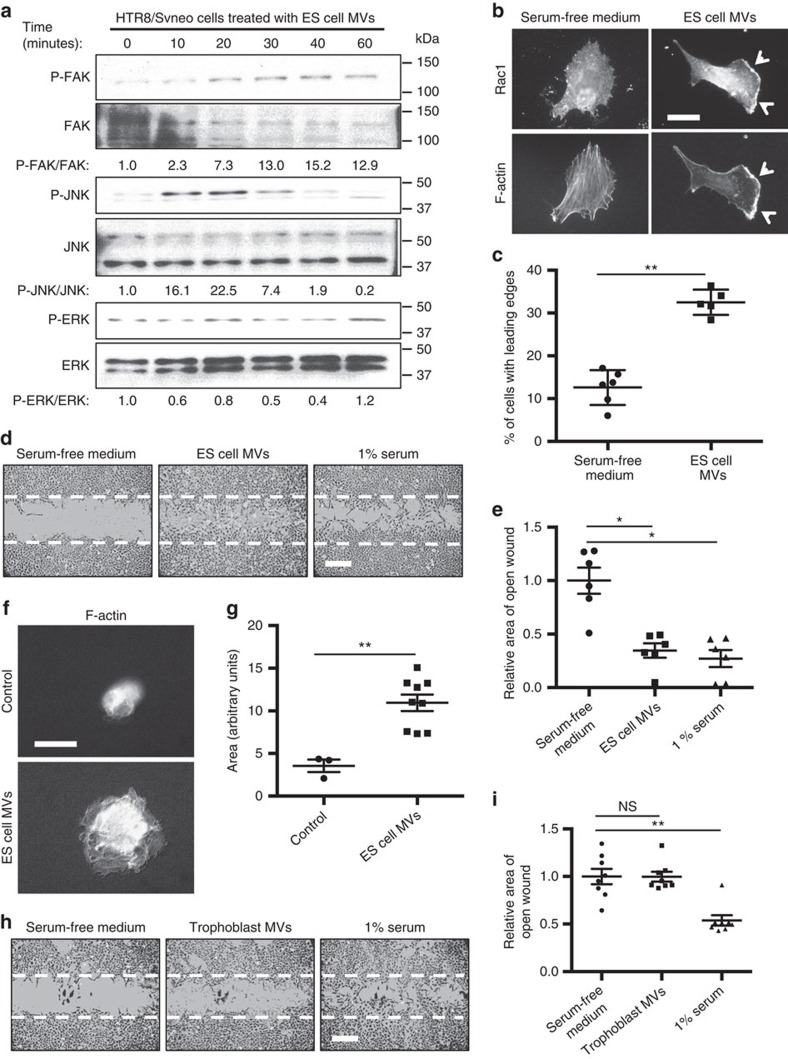
Trophoblasts treated with MVs from ES cells acquire a migratory phenotype. (**a**) Immunoblot of serum-starved HTR8/SVneo trophoblasts treated with serum-free medium supplemented with MVs from ES cells for the indicated lengths of time. The ratio of each phospho-protein/total protein pair examined was determined and included on the blots. (**b**) HTR8/SVneo trophoblasts were incubated with serum-free medium supplemented without (Serum-free medium) or with MVs isolated from ES cells (ES cell MVs) for 1 h, at which time the cells were stained for Rac1 and F-actin using rhodamine-conjugated phalloidin. Images of the cells are shown. Arrowheads denote leading edges. Scale bar, 20 μm. (**c**) The percentages of cells in **b** with leading edges were determined. (**d**) Images of wound closure assays performed on HTR8/SVneo cells cultured in serum-free medium supplemented without (Serum-free medium) or with either MVs from ES cells (ES cell MVs) or 1% serum. The dashed line indicates the width of the original wound. Scale bar, 250 μm. (**e**) The assays in **d** were quantified and plotted as the relative area of open wound. (**f**) E3.5 blastocysts were harvested and placed in dishes containing blastocyst culturing medium supplemented without (Control) or with ES cell MVs. Two days later, the blastocysts were stained with rhodamine-conjugated phalloidin and visualized by fluorescence microscopy. Images of the blastocysts are shown. Scale bar, 100 μm. (**g**) The assays in **f** were quantified and graphed as relative area of blastocyst outgrowth. (**h**) Images of wound closure assays performed on HTR8/SVneo cells cultured in serum-free medium supplemented without (Serum-free medium), or with either trophoblast MVs or 1% serum. The dashed line indicates the width of the original wound. Scale bar, 250 μm. (**i**) The assays in **h** were quantified and plotted as the relative area of open wound. All values shown are presented as mean±s.e.m. (*n*≥3 independent experiments for each assay). Differences were analysed using Student's *t*-test; *P<0.05, **P<0.01, NS, not significant.

Because MVs from aggressive cancer cells activate signalling activities in different types of recipient cells^3^,^5^,^15^,^30^, we examined whether MVs derived from ES cells could similarly affect trophoblasts, and in particular, if they were capable of activating the same protein kinases that were stimulated by the CM from ES cells. Indeed, we found that ES cell-shed MVs induced a time-dependent phosphorylation of FAK (P-FAK) and JNK (P-JNK), similar to when trophoblasts were incubated with the CM from ES cells (Fig. 2a).

We then examined whether MVs generated by ES cells stimulated trophoblasts to form leading edges, a hallmark of polarized cell migration. Trophoblasts placed in serum-free medium, either lacking or supplemented with ES cell-derived MVs for 1 h, were stained for filamentous actin (F-actin) and Rac1, two proteins that localize at the leading edges of migrating cells^33^,^34^. The resulting fluorescence microscopy images of the trophoblasts cultured in serum-free medium showed that Rac1 was localized throughout the cell, with F-actin forming well-defined stress fibers (Fig. 2b, left panels). However, the MV-treated trophoblasts exhibited a polarized morphology, with Rac1 being localized to discrete regions along their plasma membranes (Fig. 2b, top right panel), and were largely devoid of detectable stress fibers. F-actin was aligned along the leading edges of the cells (Fig. 2b, bottom right panel). Nearly 35% of the trophoblasts treated with ES cell-derived MVs showed well-defined leading edges, representing a greater than twofold increase compared with the untreated control cells (Fig. 2c).

The ability of ES cell MVs to induce trophoblasts to form leading edges was accompanied by an enhanced migration as measured in wound healing assays, which was comparable to the stimulation obtained with serum (Fig. 2d,e). We then set out to determine whether MVs from ES cells promoted blastocyst outgrowth, which is thought to reflect the implantation process and the ability of the trophectoderm in the blastocyst stage embryo to attach to a culture dish and migrate^35^. E3.5 blastocysts isolated from mice were randomly divided into two groups. One group was cultured in a dish containing blastocyst culture medium, while the second group was cultured in the same medium supplemented with MVs collected from ES cells. Figure 2f shows representative images of blastocysts that were cultured in medium lacking (Control, left) or containing ES cell MVs (right) for 48 h, at which point they were stained with rhodamine-conjugated phalloidin to label F-actin and visualized by fluorescence microscopy. The trophoblasts in the blastocysts cultured with MVs showed enhanced migration, forming outgrowths that were at least threefold greater in area than their control counterparts (Fig. 2g).

We then examined whether MVs generated by ES cells were uniquely capable of influencing trophoblast migration. We discovered that the HTR8/SVneo trophoblasts generated MVs of similar sizes and amounts as ES cells, as read out by DLS (Supplementary Fig. 3c) and immunoblot analysis using the MV marker flotillin-2 (Supplementary Fig. 3d). MVs could also be detected on the surfaces of trophoblasts stained with the FM1-43fx plasma membrane dye (Supplementary Fig. 3e). However, MVs generated by HTR8/SVneo trophoblasts, when collected and added back to cultures of trophoblasts, were unable to promote their own migration (Fig. 2h,i). Trophoblast MVs also failed to change the amount of blastocyst attachment and outgrowth compared with control outgrowths (Supplementary Fig. 3f,g).

### Laminin and fibronectin mediate the effects of ES cell MVs

**Figure 3:**
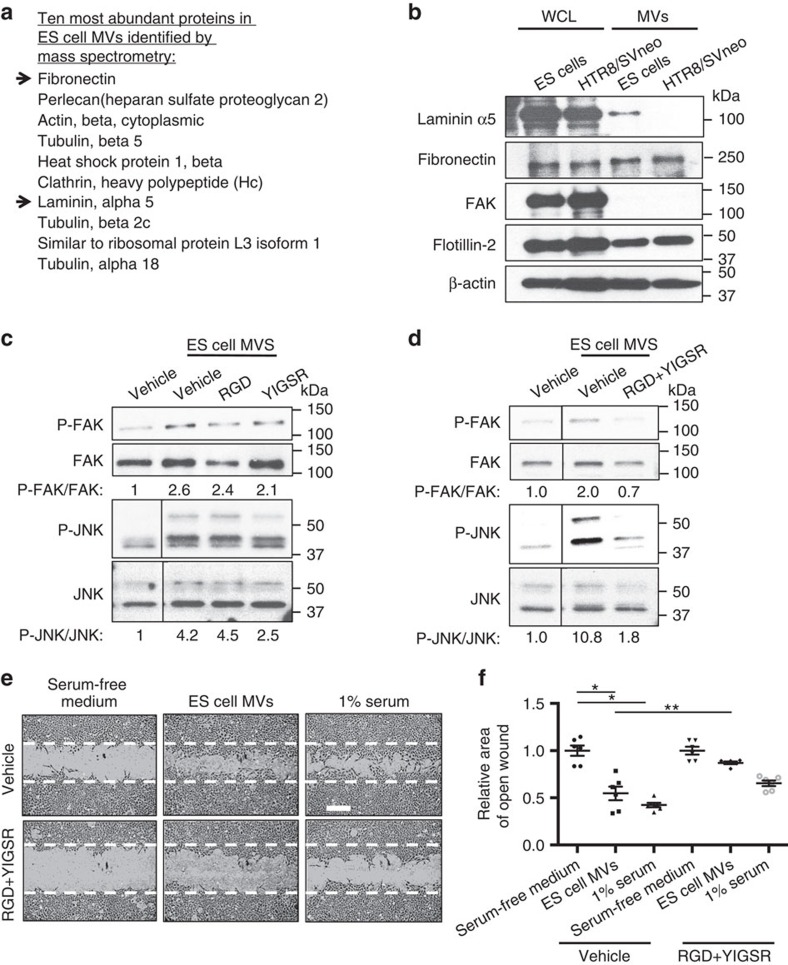
Fibronectin and laminin mediate MV-promoted trophoblast migration. (**a**) Mass spectrometry was performed on MVs isolated from ES cells. The 10 most abundant proteins identified are listed. Arrows highlight proteins predicted to most likely mediate the migration-promoting effects of MVs. (**b**) Lysates of ES cells (WCL), and the MVs that they generated (MVs), were immunoblotted for fibronectin, laminin α5, the MV marker flotillin-2, the cytosolic-specific protein FAK, and β-actin, as a loading control. (**c**,**d**) Serum-starved HTR8/SVneo trophoblasts were treated with PBS (Vehicle) or with either (**c**) the fibronectin inhibitory peptide RGD or the laminin inhibitory peptide YIGSR or (**d**) a combination of the two inhibitors. The cells were then stimulated with ES cell MVs for ∼30 min before being lysed and immunoblotted for phosphorylated and total FAK and JNK. The ratio of each phospho-protein/total protein pair examined was determined and included on the blots. Note only the combination of RGD and YIGSR blocked integrin-mediating phosphorylation of FAK and JNK. (**e**) Images were taken of wound closure assays performed on HTR8/SVneo cells cultured in serum-free medium supplemented with the vehicle (top panels) or the RGD- and YIGSR-inhibitory peptides (bottom panels). Each culturing condition was further treated without (Serum free medium) or with either MVs from ES cells (ES cell MVs) or 1% serum. The dashed line indicates the width of the original wound. Scale bar, 250 μm. (**f**) The assays in **e** were quantified and plotted as the relative area of open wound. All values shown are presented as mean±s.e.m. (*n*≥3 independent experiments for each assay). Differences were analysed using Student's *t*-test; *P<0.05, **P<0.01.

Since MVs isolated from ES cells are capable of rapidly (within 1 h) activating signalling events within recipient trophoblasts, as well as inducing leading edge formation, we suspected that proteins associated with the MVs were responsible for stimulating these outcomes, rather than transferred DNA or RNA transcripts, as phenotypic changes involving transcription and/or translation typically require several hours to occur. Therefore, a proteomic analysis was performed to identify the protein cargo of MVs isolated from the E14tg2a.4 ES cell line. A large number of proteins were detected (Supplementary Data 1), with the 10 most abundant being listed in Fig. 3a. The extracellular matrix proteins fibronectin and laminin α5 were especially noteworthy, given that fibronectin was previously shown to be essential for the growth- and survival-promoting activity of MVs from aggressive cancer cell lines^3^, whereas laminin α5 knockout mice exhibited placental abnormalities^36^. These two extracellular matrix proteins were recently found to be expressed in the blastocyst at the time of implantation^37^. We confirmed the presence of both proteins in MVs derived from ES cells by immunoblot analysis (Fig. 3b, top two panels, first and third lanes). While fibronectin was present in MVs derived from HTR8/SVneo trophoblasts, laminin α5 was not (Fig. 3b, top two panels, third and fourth lanes), despite the fact that trophoblasts and ES cells express similar amounts of laminin α5 (Fig. 3b, top two panels, first two lanes). Fibronectin and laminin function as ligands that activate receptors expressed by trophoblasts^38^, with the α5β1 integrin complex serving as the receptor for fibronectin^39^, while laminin binds to the 67 kDa laminin receptor, which acts as an integrin co-receptor for integrin α6 (ref. 40). Moreover, fibronectin- and laminin-induced integrins signal through FAK and JNK^41^,^42^. Both of these protein kinases were activated in trophoblasts treated with MVs from ES cells, and are required for trophoblast migration, that is, trophoblasts incubated with either a JNK or FAK inhibitor failed to migrate in wound closure assays (Supplementary Fig. 4a–d). Consistent with these findings, treating-isolated blastocysts with these same inhibitors also prevented their attachment and outgrowth onto a substrate (Supplementary Fig. 4e).

To determine whether MV-associated fibronectin and laminin were responsible for activating FAK and JNK, we examined the effects of blocking their ability to engage their respective receptors on the surfaces of trophoblasts. The interaction of fibronectin with α5β1 integrins was disrupted using the RGD peptide^43^, while the binding of laminin to its receptor was blocked using the YIGSR peptide^44^. Treating HTR8/SVneo trophoblasts with the RGD peptide alone, before incubating them with MVs isolated from ES cells, failed to prevent MV-induced FAK and JNK activation (Fig. 3c, first and third panels). Similarly, treatment with the YIGSR peptide was not sufficient to block MV-stimulated FAK activation (Fig. 3c, top panel) and caused only a modest reduction of JNK activity (Fig. 3c, third panel). However, when the two inhibitors were used concurrently, they effectively blocked the ability of MVs to activate these protein kinases (Fig. 3d, first and third panels).

Confirmation that ES cell MVs require fibronectin and laminin to promote trophoblast migration was then obtained from wound closure assays in which the RGD and YIGSR peptides were added together to trophoblasts that were either cultured in serum-free medium, or treated with medium containing ES cell MVs or serum. The addition of these peptides, in combination, but not when added individually, efficiently inhibited MV-promoted trophoblast migration, reducing it to a level comparable to that observed in serum-starved conditions (Fig. 3e,f; Supplementary Fig. 5a–d). In contrast, however, the exposure of trophoblasts to the combination of purified forms of fibronectin and laminin stimulated their migration to an extent similar to that obtained with full serum treatment (Supplementary Fig. 5e,f). Taken together, these findings indicate that the extracellular matrix proteins fibronectin and laminin are essential for the ability of ES cell MVs to activate integrin-mediated signalling events necessary for promoting trophoblast migration.

### ES cell-derived MVs promote blastocyst implantation

**Figure 4:**
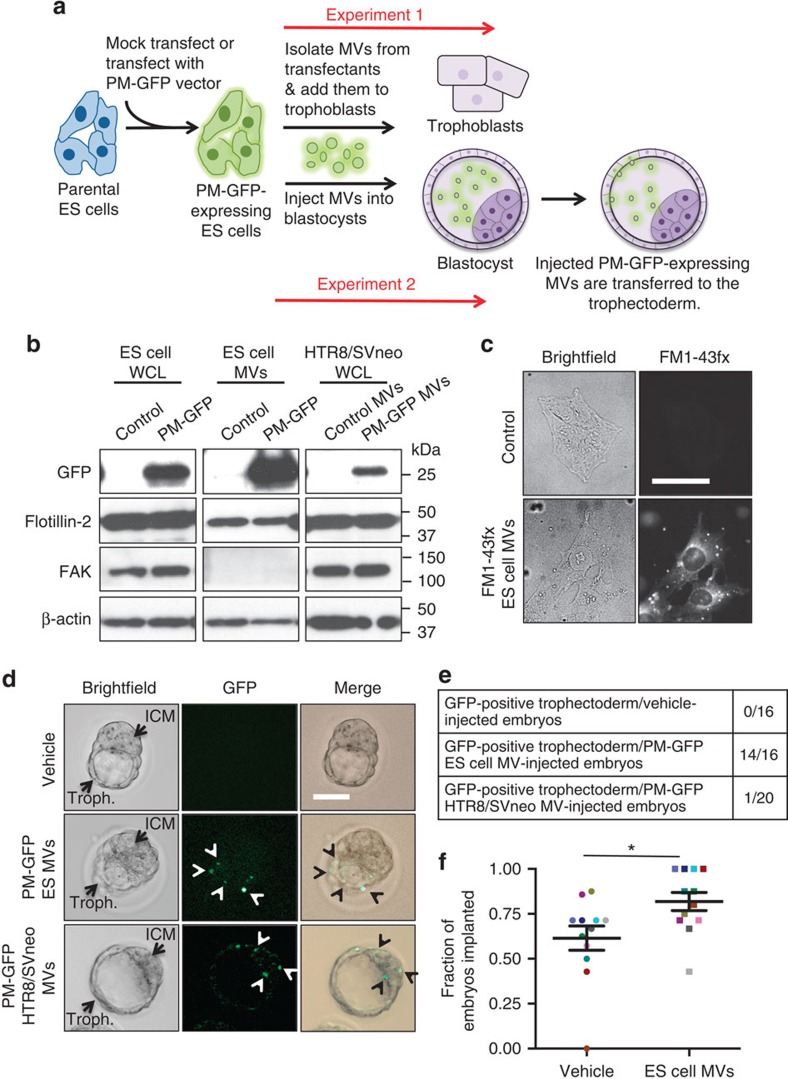
MVs from ES cells are transferred to trophoblasts and promote implantation. (**a**) Description of experiments (Experiment 1 and 2) conducted to demonstrate that MV cargo is transferred to trophoblasts. (**b**) Experiment 1; MVs from mock-transfected ES cells (Control) or ES cells ectopically expressing PM-GFP were isolated and then either lysed or resuspended in medium. Lysates of the transfectants (ES cell WCL, lanes 1 and 2), and their MVs (ES cell MVs, lanes 3 and 4), were immunoblotted for GFP, the MV marker flotillin-2, the cytosolic-specific marker FAK, and β-actin, as a loading control. The MVs resuspended in medium were added to trophoblasts for 3 h, at which time the cells were extensively washed, lysed, and immunoblotted as indicated (HTR8/SVneo WCL, lanes 5 and 6). (**c**) Trophoblasts treated for 3 h with basal medium (Control) or ES cell CM treated with FM1-43fx plasma membrane dye before being subjected to the MV isolation procedure were fixed and visualized by brightfield and fluorescence microscopy. The scale bar is 50 μm. (**d**) Experiment 2; PBS (Control, top panels), or MVs derived from either ES cells (middle panels) or HTR8/SVneo trophoblasts (bottom panels) ectopically expressing PM-GFP were injected into E3.5 blastocysts. Three hours later bright field (left panels), fluorescence (GFP, centre panels), and merged images (right panels) of the blastocysts were taken. The ICM and trophectoderm (Troph.) in the blastocysts are labelled, as is the GFP signal detected within the blastocysts (arrowheads). Scale bar, 50 μm. (**e**) The ratio of blastocysts in **d** with detected levels of GFP signal in their trophectoderm. (**f**) E3.5 blastocysts were injected with either a PBS vehicle control or MVs from ES cells. The vehicle alone-injected blastocysts were surgically placed into the right uterine horn of a surrogate mouse, while the blastocysts injected with MVs were placed in the left uterine horn of the same mouse. Three days later, the uteri were harvested and the rate of implantation for each condition/mouse was determined and plotted as color-coded pairs. A total of 168 blastocysts and 12 surrogate mice were used in four independent experiments. Differences were analyzed using Wilcoxon signed-rank test; *P≤0.05.

We next wanted to determine whether the MV-mediated intercellular communication between ES cells and trophoblasts occurs within blastocysts. To demonstrate that this is the case, we took advantage of earlier work by our laboratory showing that plasma membrane-targeted green fluorescent protein (PM-GFP) was capable of being incorporated into MVs generated by cancer cells and then transferred via MVs to recipient cells^3^. Duplicate sets of parental E14tg2a.4 ES cells were either mock transfected (Control) or transfected with PM-GFP (Fig. 4a, Experiment 1). The MVs collected from one set of the transfected cells showed a significant incorporation of PM-GFP (Fig. 4b, lanes labelled ES cell MVs, top panel), similar to what we had previously observed for MVs isolated from the highly aggressive MDA-MB231 breast cancer cell line expressing this fusion protein^45^. MVs isolated from either the second set of mock-transfected ES cells (Control), or from the ES cells ectopically expressing PM-GFP, were then added to cultures of trophoblasts for 3 h, at which time the trophoblasts were extensively washed before being immunoblotted with a GFP antibody. PM-GFP was clearly detectable in the trophoblasts that had been incubated with the PM-GFP-labelled MVs (Fig. 4b, lanes labelled HTR8/SVneo WCL, top panel).

To further confirm the uptake of ES cell MVs by trophoblasts, basal medium (Control) or ES cell CM was treated with FM1-43fx plasma membrane dye before being subjected to the MV isolation procedure. The MV preparations were incubated with trophoblasts for 3 h, at which time the cells were visualized by brightfield and fluorescence microscopy. While the control-treated trophoblasts failed to exhibit any fluorescence emission (Fig. 4c, panels labelled Control), nearly all of the trophoblasts treated with MVs labelled with FM1-43fx showed detectectable fluorescence (Fig. 4c, panels labelled FM1-43fx ES cell MVs).

We then examined whether MVs derived from ES cells can transfer their cargo to the layer of trophoblasts surrounding the ICM, called the trophectoderm (Fig. 4a, Experiment 2). MVs derived from ES cells ectopically expressing PM-GFP (Fig. 4b, lanes labelled ES cell MVs, top panel) or vehicle alone were injected into the cavity of embryonic day 3.5 (E3.5) blastocysts, and then the blastocysts were visualized by microscopy ∼3 h later. Figure 4d shows bright field images (left panels), fluorescent images (middle panels) and merged images (right panels) of three blastocysts: one injected with vehicle (top panels), one with ES cell MVs expressing PM-GFP (middle panels), and another with HTR8/SVneo trophoblast MVs expressing PM-GFP (bottom panels). GFP signal was not detected in any of the blastocysts injected with the vehicle alone (Fig. 4d, top panel and Fig. 4e). In contrast, GFP signal could be clearly detected in discrete regions along the trophectoderm in 14/16 of the blastocysts injected with ES cell MVs (Fig. 4d, middle panels and Fig. 4e), suggesting that MVs shed from ES cells were indeed capable of being transferred to the trophectoderm. This was not the case, however, for the blastocysts injected with HTR8/SVneo trophoblast MVs, as only 1/20 of these blastocysts had detectable levels of GFP signal along the trophectoderm. Rather, these blastocysts tended to show GFP signal in their ICMs (Fig. 4d, bottom panels).

In addition, the MV-mediated intercellular communication between ES cells and trophoblasts positively impacted the ability of the trophoblasts to undergo implantation. Specifically, we found that injecting E3.5 blastocysts with MVs isolated from ES cells, before surgically placing them into the uteri of surrogate mice, gave rise to a statistically significant enhancement in the likelihood that the blastocysts implanted when compared to blastocysts injected with vehicle alone (Fig. 4f). The raw data and statistical analysis of these results are also included (Supplementary Table 1).

## Discussion

It is becoming increasingly appreciated that EVs provide an important mechanism for cell–cell communication^6^,^46^. Thus far, this has been particularly well demonstrated within the context of tumorigenesis, where EVs shed by cancer cells can be transferred to (recipient) cells within the tumour microenvironment and alter their behaviour in ways that drive cancer progression^3^,^5^,^12^,^14^,^15^,^30^. However, in recent years, key roles for EVs have also been identified in a number of different physiological contexts, including development and even the earliest stages of embryogenesis^47^,^48^. For example, EVs released by uterine cells are being examined for their potential roles in influencing embryo implantation and the establishment of a successful pregnancy^49^,^50^,^51^, and various studies have demonstrated that blastocysts themselves also generate and shed EVs^52^,^53^,^54^,^55^. Moreover, it has been shown that ES cells are capable of generating EVs (that is, exosomes and MVs), which have been suggested to enhance the survival and intrinsic ‘stem-ness' of progenitor cells, as well as transfer their cargo and alter gene expression in recipient fibroblasts^1^,^2^,^56^,^57^. Still, we have barely scratched the surface in understanding the full breadth of roles played by EVs in stem cell biology.

**Figure 5:**
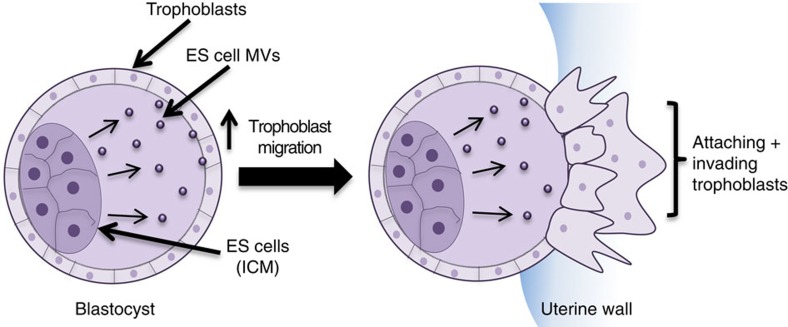
Diagram showing how ES cells communicate with trophoblasts to promote embryo implantation. In the blastocyst stage embryo, the ES cells reside in the ICM and are surrounded by trophoblasts (left image). While it is known that the trophoblasts in the blastocyst respond to signals emanating from the mother to migrate and invade into the uterus (implantation), we have discovered that ES cells also contribute to this process by generating MVs (right image). The MVs are capable of activating signalling events in trophoblasts that enhance their ability to migrate.

We now show that ES cells generate and shed a class of EVs known as MVs that are capable of impacting one of the earliest and most important steps in pregnancy, implantation. Specifically, ES cell MVs make use of the extracellular matrix proteins fibronectin and laminin α5, that are aligned along their outer surfaces, to bind integrin α5β1 and the laminin receptor on trophoblasts. These interactions between ES cell MVs and trophoblasts activate FAK and JNK, within the trophoblasts, thereby promoting trophoblast migration. We further show that ES cell MVs transfer their contents to trophoblasts, as well as directly interact with the trophectoderm layer in the blastocyst state embryo, and when injected into blastocysts, they markedly enhance the ability of blastocysts to undergo implantation (Fig. 5). Thus far, the vast majority of the studies examining the mechanisms that underlie implantation have focused on the communication events that occur between maternal cells and trophoblast cells^19^,^20^,^58^,^59^,^60^,^61^. Therefore, to the best of our knowledge, the findings reported here provide the first demonstrations that the embryo can send signals which directly promote trophoblast migration and influence the implantation process. It is worth noting that trophoblasts, like ES cells, produce MVs. However, unlike what we have found for ES cell MVs, trophoblast-derived MVs appear to be incapable of influencing trophoblast migration. This is most likely due to the fact that trophoblast MVs contain significantly less laminin α5 compared with ES cells MVs, thereby making them ineffective in stimulating the signalling activities necessary for promoting trophoblast migration.

The early stages of pregnancy, especially implantation, are vital for successful pregnancies. Complications that arise during implantation are a major cause of infertility, but they can also lead to other serious conditions, such as preeclampsia^19^. Infertility is a major health issue, and *in vitro* fertilization is costly and has a relatively low success rates (<30%)^62^. One of the main causes of failure during *in vitro* fertilization is the inability of the embryo to implant^62^,^63^. Our findings showing that ES cell MVs enhance implantation rates now raise some exciting possibilities regarding their potential use in therapeutic applications for promoting the natural ability of embryos to successfully implant and establish a pregnancy.

## Methods

### Antibodies and reagents

Antibodies that recognize the total or phosphorylated forms of JNK (catalogue nos 9252 and 4668), FAK (catalogue nos 3285 and 3283), ERK1/2 (catalogue nos 9102 and 9106) and flotillin-2 (catalogue no. 3436) were from Cell Signaling Technology and used at 1:2,000. The GFP (catalogue no. SC-8334) and Oct3/4 (catalogue no. SC-5279) antibodies were from Santa Cruz Biotechnology and used at 1:500, the Ran (catalogue no. 610340) antibody was from BD Transduction Laboratories and used at 1:1,000, while the Nanog (catalogue no. ab80892) antibody was from Abcam and used at 1:700. The fibronectin (catalogue no. F6140), β-actin (catalogue no. A5316) and laminin α5 (catalogue no. WH0003911M1) antibodies used at 1:1,000, as well as GRGDSP (RGD) peptide and YIGSR peptide were from Sigma-Aldrich. The 0.22 μm Steriflip filters, centrifugal filters, Rac1 antibody (catalogue no. 05-389) used at 1:250, FAK inhibitor III, PD98059, SP600125, embryonic stem (ES) cell-qualified fetal bovine serum (FBS), LIF and M2 medium were from Millipore. The PM-GFP vector was a gift from Tobias Meyer (Addgene plasmid #21213). The rhodamine-conjugated phalloidin, Oregon green 488 goat anti-mouse secondary antibody, FM1-43fx plasma membrane dye, Lipofectamine, as well as all other tissue culture reagents were from Life Technologies.

### Cell culture

E14tg2a.4 mouse ES cells were cultured on gelatin-coated dishes in Glasgow's Minimal Essential Medium supplemented with 1 mM sodium pyruvate, non-essential amino acids, 55 μM β-mercaptoethanol, 15% ES cell-qualified FBS and 1,000 U ml^−1^ LIF. HTR8/SVneo trophoblast cells, which were a gift from Charles Graham (Queen's University, Kingston, ON), were cultured in RPMI-1640 medium supplemented with 5% FBS.

### Concentration of CM and MV isolation

One 150-mm dish of nearly confluent ES cells (∼4.0 × 10^7^ cells) was rinsed several times with PBS and incubated in serum- and LIF-free medium for 5 h. The CM was removed from the cells, and first centrifuged at 300*g* for 10 min to pellet intact cells, and again at 3,000*g* for 20 min to remove cell debris. The partially clarified medium was concentrated to 1 ml using a centrifugal filter with a nominal molecular weight limit of 10 kDa and then added to recipient cells. As a control for the experiments involving CM, an equivalent volume of base medium (that is, ES cell medium lacking serum and LIF) was concentrated and then added to recipient cells. To isolate MVs, the CM from two nearly confluent 150-mm dishes of ES cells (∼8.0 × 10^7^ cells) was partially clarified as described above, filtered through a 0.22-μm Steriflip filter unit (Millipore), and then washed with 10 ml of PBS. If being used in biological assays, the MVs retained by the filter were resuspended in 1.5 ml of serum-free medium, treated with inhibitors or peptides if indicated, and added to ∼2.0 × 10^5^ trophoblasts. As a control for all experiments involving MVs, the same medium that the MVs were resuspended in was added to another set of recipient cells. When used to generate lysates, the MVs were lysed with mammalian lysis buffer (25 mM Tris, 100 mM NaCl, 1% Triton X-100, 1 mM EDTA, 1 mM DTT, 1 mM NaVO_4_, 1 mM β-glycerol phosphate, 1 μg ml^−1^ aprotinin and 1 μg ml^−1^ leupeptin) directly off the filter.

### Signalling experiments in trophoblasts

Serum-starved trophoblasts were trypsinized, counted, and ∼2.0 × 10^5^ cells were placed into two different tubes. The cells were centrifuged at 300*g* for 5 min, and the pelleted cells were then resuspended in serum-free medium supplemented without or with either CM or MVs isolated from ES cells. Following the indicated lengths of incubation, the cells were re-pelleted and lysed in mammalian lysis buffer. For the MV transfer experiments, MVs isolated from ES cells expressing the vector alone or PM-GFP or ES cell MVs treated with FM1-43fx were added to ∼2.0 × 10^5^ adherent trophoblasts for 3 h, at which time the trophoblasts were lysed in mammalian lysis buffer or fixed with 3.7% formaldehyde in PBS for 20 min, as indicated.

### Immunoblot analysis

Protein concentrations of MV and cell lysates were determined using the Bio-Rad DC protein assay. For the immunoblots where MVs were analysed, 5 μg of MV lysate and WCL was used. For the immunoblots where the signalling activity of various proteins was determined, 15 μg of each lysate was used. Equal concentrations of lysates were resolved by SDS–PAGE and then transferred to PVDF membranes. The membranes were incubated with the indicated primary antibodies diluted in 20 mM Tris, 135 mM NaCl, and 0.02% Tween 20 (TBST). Primary antibodies were detected with HRP-conjugated secondary antibodies (Cell Signaling Technology), followed by exposure to ECL reagent. Where indicated, changes in protein phosphorylation were determined by normalization to total protein levels using ImageJ. In the cases where P-JNK and P-ERK levels were determined, both isoforms of JNK (JNK1/2: 46 and 54 kDa) and ERK (ERK1/2: 42 and 44 kDa) were used. All images shown in the manuscript were cropped, but images of the full, uncropped blots used in each figure can be viewed in Supplementary Fig. 6.

### Mass spectrometry

Lysates of MVs isolated from E14tg2a.4 ES cells (30 μg) were resolved by SDS–PAGE and then stained with a Colloidal Blue Staining kit. The proteins were excised from the gel and trypsin-digested. Cornell's Proteomics Facility analysed the resulting peptide fragments using a triple quadrupole linear ion trap (4000 Q Trap) online LC/MS/MS system (Applied Biosystems/MDS Sciex) or the Synapt HDMS system (Waters). Proteins were identified by performing peptide alignment searches using the NCBI mouse RefSeq protein database.

### Fluorescence microscopy

To visualize MVs on the surfaces of ES cells and trophoblasts, cultures of cells were incubated with 5 μg ml^−1^ FM1-43fx plasma membrane dye diluted in PBS for 1 min, fixed in ice-cold 3.7% formaldehyde, and then rinsed with PBS before being analysed by fluorescence microscopy. To visualize leading edges on trophoblasts, cultures of cells that had been treated as indicated were fixed with 3.7% formaldehyde, permeabilized with 0.1% Triton X-100, and then blocked with 10% bovine serum albumin. The cells were incubated with a Rac1 antibody, washed, and then incubated with an Oregon Green 488-conjugated secondary antibody, rhodamine-conjugated phalloidin to stain F-actin, and DAPI to label nuclei. The cells were then visualized by fluorescence microscopy. All images of the cells were captured and processed using IPLABS software.

### Dynamic light scattering

To determine the sizes of EVs by DLS analysis, CM or MV preparations were loaded into microcuvettes and analysed for particle size using a Zetasizer (Malvern Nano ZS; He-Ne laser; 173° backscattered light detection). At least three independent experiments were performed for each condition analysed, and each result shown represents an average of three runs, with at least five measurements being taken per run.

### Transmission electron microscopy

Five microlitres of a MV preparation resuspended in PBS were added to a carbon-coated, 300-mesh copper grid and then stained with 1.75% uranyl acetate. Once dry, the samples were imaged using the FEI T12 Spirit 120 kV field emission transmission electron microscopy at Cornell's Center for Materials Research (CCMR), supported by NSF MRSEC award number: NSF DMR-1120296.

### Wound closure (migration) assays

Wounds were struck through confluent monolayers of serum-starved trophoblasts using a pipet tip. The cells were rinsed with PBS, placed in the indicated culturing conditions, and allowed to migrate for 12 h. The cells were then fixed in 3.7% formaldehyde. The extent of wound closure for each condition was imaged and plotted.

### Blastocyst collection and outgrowth assays

Experiments were performed as described previously^35^. In brief, post-coital day 3.5 female BALB/c and B6-2J mice between 8 and 20 weeks old (Jackson Laboratory) were killed and their uteri removed. The uteri were flushed with M2 medium to remove the blastocysts, which were then cultured on gelatin-coated dishes in Dulbecco's Minimal Essential Medium supplemented with 10% FBS, non-essential amino acids, 55 μM β-mercaptoethanol, 100 U ml^−1^ penicillin, and 100 μg ml^−1^ streptomycin and treated as indicated. Once the blastocysts attached to the dish and spread, they were fixed and stained with rhodamine-conjugated phalloidin. Images of the blastocysts were captured and processed using IPLABS software, and the areas of blastocyst outgrowth were determined using ImageJ.

### Microinjection of blastocysts and determination of implantation rates

Isolated blastocysts were injected with MVs derived from ES cells or HTR8/SVneo trophoblasts ectopically expressing PM-GFP or vehicle (PBS) by Cornell's Induced Pluripotent Stem Cell Core Facility, supported by NYSDOH contract no. C029155. The blastocysts were incubated in blastocyst culturing medium for ∼3 h, at which point they were visualized using light and fluorescence microscopy. For the *in vivo* studies, blastocysts injected with MVs derived from parental ES cells were surgically placed in the left uterine horn of B6-2J pseudo-pregnant recipient mouse between 8 and 20 weeks old (Jackson Laboratory), while an equal number of blastocysts injected with vehicle alone (PBS) were placed in the right uterine horn of the same mouse. Three days later the mouse was killed, its uterus was removed and the percentages of embyros that implanted for each condition was determined. This experiment was carried out on four separate occasions, using a total of 168 blastocysts (84 for each condition) and 12 mice as embryo transfer recipients. All experiments involving mice were carried out in accordance with the Cornell University Institutional Animal Care and Use Committee (IACUC).

### Statistical analysis

All experiments were performed a minimum of three independent times. Wound closure assays and western blots were quantified using ImageJ. Many of the results were presented as scatter plots with mean and s.e.m. plotted using GraphPad Prism 6. Student's *t*-tests were performed to assess statistical significance in all cases, except for the experiments involving the comparison of implantation rates, for which a Wilcoxon signed-rank test was used. *P*-values ≤0.05 were considered significant and indicated with asterisks, as follows: *P≤0.05, **P<0.01, ***P<0.001, NS, not significant.

### Data availability

The authors declare that the data supporting the findings of this study are available within the article and its Supplementary Information Files.

## Additional information

**How to cite this article:** Desrochers, L. M. *et al.* Microvesicles provide a mechanism for intercellular communication by embryonic stem cells during embryo implantation. *Nat. Commun.* 7:11958 doi: 10.1038/ncomms11958 (2016).

## Supplementary Material

Supplementary Information Supplementary Figures 1-6, Supplementary Table 1

Supplementary Data Proteins identified in MVs isolated from ES cells using mass spectrometry

Peer Review File Proteins identified in MVs isolated from ES cells using mass spectrometry

## References

[b1] Ratajczak J. *et al.* Embryonic stem cell-derived microvesicles reprogram hematopoietic progenitors: evidence for horizontal transfer of mRNA and protein delivery. Leukaemia 20, 847–856 (2006). 10.1038/sj.leu.2404132 16453000

[b2] Yuan A. *et al.* Transfer of microRNAs by embryonic stem cell microvesicles. PLoS ONE 4, e4722 (2009). 19266099 10.1371/journal.pone.0004722 PMC2648987

[b3] Antonyak M. A. *et al.* Cancer cell-derived microvesicles induce transformation by transferring tissue transglutaminase and fibronectin to recipient cells. Proc. Natl. Acad. Sci. USA 108, 4852–4857 (2011). 21368175 10.1073/pnas.1017667108 PMC3064359

[b4] Rak J. Microparticles in cancer. Semin. Thromb. Hemost. 36, 888–906 (2010). 21049390 10.1055/s-0030-1267043

[b5] Muralidharan-Chari V., Clancy J. W., Sedgwick A. & D'Souza-Schorey C. Microvesicles: mediators of extracellular communication during cancer progression. J. Cell Sci. 123, 1603–1611 (2010). 20445011 10.1242/jcs.064386 PMC2864708

[b6] Raposo G. & Stoorvogel W. Extracellular vesicles: exosomes, microvesicles, and friends. J. Cell Biol. 200, 373–383 (2013). 23420871 10.1083/jcb.201211138 PMC3575529

[b7] Thery C., Zitvogel L. & Amigorena S. Exosomes: composition, biogenesis and function. Nat. Rev. Immunol. 2, 569–579 (2002). 12154376 10.1038/nri855

[b8] Antonyak M. A. & Cerione R. A. Cancer Cell Signaling Methods and Protocols ed. Robles-Flores M. 147–173Springer (2014).

[b9] Pan B.-T. & Johnstone R. M. Fate of the transferrin receptor during maturation of sheep reticulocytes *in vitro*: selective externalization of the receptor. Cell 33, 967–978 (1983). 6307529 10.1016/0092-8674(83)90040-5

[b10] Ratajczak J., Wysoczynski M., Hayek F., Janowska-Wieczorek A. & Ratajczak M. Z. Membrane-derived microvesicles: important and underappreciated mediators of cell-to-cell communication. Leukaemia 20, 1487–1495 (2006). 10.1038/sj.leu.2404296 16791265

[b11] Mathivanan S. & Simpson R. J. ExoCarta: a compendium of exosomal proteins and RNA. Proteomics 9, 4997–5000 (2009). 19810033 10.1002/pmic.200900351

[b12] Al-Nedawi K. *et al.* Intercellular transfer of the oncogenic receptor EGFRvIII by microvesicles derived from tumour cells. Nat. Cell Biol. 10, 619–624 (2008). 18425114 10.1038/ncb1725

[b13] Raposo G. *et al.* B lymphocytes secrete antigen-presenting vesicles. J. Exp. Med. 183, 1161–1172 (1996). 8642258 10.1084/jem.183.3.1161 PMC2192324

[b14] Peinado H. *et al.* Melanoma exosomes educate bone marrow progenitor cells toward a pro-metastatic phenotype through MET. Nat. Med. 18, 883–891 (2012). 22635005 10.1038/nm.2753 PMC3645291

[b15] Skog J. *et al.* Glioblastoma microvesicles transport RNA and proteins that promote tumour growth and provide diagnostic biomarkers. Nat. Cell Biol. 10, 1470–1476 (2008). 19011622 10.1038/ncb1800 PMC3423894

[b16] Costa-Silva B. *et al.* Pancreatic cancer exosomes initiate pre-metastatic niche formation in the liver. Nat. Cell Biol. 17, 816–826 (2015). 25985394 10.1038/ncb3169 PMC5769922

[b17] Hoshino A. *et al.* Tumour exosome integrins determine organotropic metastasis. Nature 527, 329–335 (2015). 26524530 10.1038/nature15756 PMC4788391

[b18] EL Andaloussi S., Mager I., Breakefield X. O. & Wood M. J. A. Extracellular vesicles: biology and emerging therapeutic opportunities. Nat. Rev. Drug Discov. 12, 347–357 (2013). 23584393 10.1038/nrd3978

[b19] Norwitz E. R., Schust D. J. & Fisher S. J. Implantation and the survival of early pregnancy. N. Engl. J. Med. 345, 1400–1408 (2001). 11794174 10.1056/NEJMra000763

[b20] Red-Horse K. *et al.* Trophoblast differentiation during embryo implantation and formation of the maternal-fetal interface. J. Clin. Invest. 114, 744–754 (2004). 15372095 10.1172/JCI22991 PMC516273

[b21] Redman C. W. & Sargent I. L. Latest advances in understanding preeclampsia. Science 308, 1592–1594 (2005). 15947178 10.1126/science.1111726

[b22] Resnik R. Intrauterine growth restriction. Obstet. Gynecol. 99, 490–496 (2002). 11864679 10.1016/s0029-7844(01)01780-x

[b23] Goldin S. N. & Papaioannou V. E. Paracrine action of FGF4 during periimplantation development maintains trophectoderm and primitive endoderm. Genesis 36, 40–47 (2003). 12748966 10.1002/gene.10192

[b24] Houghton F. D. Role of gap junctions during early embryo development. Reproduction 129, 129–135 (2005). 15695607 10.1530/rep.1.00277

[b25] Graham C. H. *et al.* Establishment and characterization of first trimester human trophoblast cells with extended lifespan. Exp. Cell Res. 206, 204–211 (1993). 7684692 10.1006/excr.1993.1139

[b26] Smith A. G. *et al.* Inhibition of pluripotential embryonic stem cell differentiation by purified polypeptides. Nature 336, 688–690 (1988). 3143917 10.1038/336688a0

[b27] Sieg D. J. *et al.* FAK integrates growth-factor and integrin signals to promote cell migration. Nat. Cell Biol. 2, 249–256 (2000). 10806474 10.1038/35010517

[b28] Huang C., Rajfur Z., Borchers C., Schaller M. D. & Jacobson K. JNK phosphorylates paxillin and regulates cell migration. Nature 424, 219–223 (2003). 12853963 10.1038/nature01745

[b29] Huang C., Jacobson K. & Schaller M. D. MAP kinases and cell migration. J. Cell Sci. 117, 4619–4628 (2004). 15371522 10.1242/jcs.01481

[b30] Al-Nedawi K., Meehan B., Kerbel R. S., Allison A. C. & Rak J. Endothelial expression of autocrine VEGF upon the uptake of tumor-derived microvesicles containing oncogenic EGFR. Proc. Natl. Acad. Sci. USA 106, 3794–3799 (2009). 19234131 10.1073/pnas.0804543106 PMC2656159

[b31] Li B., Antonyak M. A., Zhang J. & Cerione R. A. RhoA triggers a specific signaling pathway that generates transforming microvesicles in cancer cells. Oncogene 31, 4740–4749 (2012). 22266864 10.1038/onc.2011.636 PMC3607381

[b32] Sung B. H., Ketova T., Hoshino D., Zijlstra A. & Weaver A. M. Directional cell movement through tissues is controlled by exosome secretion. Nat. Commun. 6, 7164 (2015). 25968605 10.1038/ncomms8164 PMC4435734

[b33] Mitchison T. J. & Cramer L. P. Actin-based cell motility and cell locomotion. Cell 84, 371–379 (1996). 8608590 10.1016/s0092-8674(00)81281-7

[b34] Waterman-Storer C. M., Worthylake R. A., Liu B. P., Burridge K. & Salmon E. D. Microtubule growth activates Rac1 to promote lamellipodial protrusion in fibroblasts. Nat. Cell Biol. 1, 45–50 (1999). 10559863 10.1038/9018

[b35] Armant D. R. Placenta and Trophoblast: Methods and Protocols Vol. 1, 35–56Humana Press (2006).

[b36] Miner J. H., Cunningham J. & Sanes J. R. Roles for laminin in embryogenesis: exencephaly, syndactyly, and placentopathy in mice lacking the laminin α5 chain. J. Cell Biol. 143, 1713–1723 (1998). 9852162 10.1083/jcb.143.6.1713 PMC2132973

[b37] Boroviak T., Loos R., Bertone P., Smith A. & Nichols J. The ability of inner-cell-mass cells to self-renew as embryonic stem cells is acquired following epiblast specification. Nat. Cell Biol. 16, 513–525 (2014). 10.1038/ncb2965 PMC4878656 24859004

[b38] Armant D. R., Kaplan H. A. & Lennarz W. J. Fibronectin and laminin promote *in vitro* attachment and outgrowth of mouse blastocysts. Dev. Biol. 116, 519–523 (1986). 3732618 10.1016/0012-1606(86)90152-1

[b39] Johansson S., Svineng G., Wennerberg K., Armulik A. & Lohikangas L. Fibronectin-integrin interactions. Front. Biosci. 2, d126–d146 (1997). 9159220 10.2741/a178

[b40] Nelson J. *et al.* The 67 kDa laminin receptor: structure, function and role in disease. Biosci. Rep. 28, 33–48 (2008). 18269348 10.1042/BSR20070004

[b41] Almeida E. A. *et al.* Matrix survival signaling from fibronectin via focal adhesion kinase to C-Jun Nh2-terminal kinase. J. Cell Biol. 149, 741–754 (2000). 10791986 10.1083/jcb.149.3.741 PMC2174844

[b42] Givant-Horwitz V., Davidson B. & Reich R. Laminin-induced signaling in tumor cells. Cancer Lett. 223, 1–10 (2005). 15890231 10.1016/j.canlet.2004.08.030

[b43] Ruoslahti E. & Pierschbacher M. D. New perspectives in cell adhesion: RGD and integrins. Science 238, 491–497 (1987). 2821619 10.1126/science.2821619

[b44] Iwamoto Y. *et al.* YIGSR, a synthetic laminin pentapeptide, inhibits experimental metastasis formation. Science 238, 1132–1134 (1987). 2961059 10.1126/science.2961059

[b45] Antonyak M. A., McNeill C. J., Wakshlag J. J., Boehm J. E. & Cerione R. A. Activation of the Ras-ERK pathway inhibits retinoic acid-induced stimulation of tissue transglutaminase expression in NIH3T3 cells. J. Biol. Chem. 278, 15859–15866 (2003). 12604597 10.1074/jbc.M300037200

[b46] Cicero Lo, A., Stahl P. D. & Raposo G. Extracellular vesicles shuffling intercellular messages: for good or for bad. Curr. Opin. Cell Biol. 35, 69–77 (2015). 26001269 10.1016/j.ceb.2015.04.013

[b47] Gross J. C., Chaudhary V., Bartscherer K. & Boutros M. Active Wnt proteins are secreted on exosomes. Nat. Cell Biol. 14, 1036–1045 (2012). 22983114 10.1038/ncb2574

[b48] Lakkaraju A. & Rodriguez-Boulan E. Itinerant exosomes: emerging roles in cell and tissue polarity. Trends Cell Biol. 18, 199–209 (2008). 18396047 10.1016/j.tcb.2008.03.002 PMC3754907

[b49] Ng Y. H. *et al.* Endometrial exosomes/microvesicles in the uterine microenvironment: a new paradigm for embryo-endometrial cross talk at implantation. PLoS ONE 8, e58502 (2013). 23516492 10.1371/journal.pone.0058502 PMC3596344

[b50] Saadeldin I. M., Oh H. J. & Lee B. C. Embryonic–maternal cross-talk via exosomes: potential implications. Stem Cells Cloning 8, 103–107 (2015). 26185458 10.2147/SCCAA.S84991 PMC4500606

[b51] Tannetta D., Dragovic R., Alyahyaei Z. & Southcombe J. Extracellular vesicles and reproduction–promotion of successful pregnancy. Cell Mol. Immunol. 11, 548–563 (2014). 24954226 10.1038/cmi.2014.42 PMC4220835

[b52] Gardiner C. F. *et al.* IVF embryos release extracellular vesicles which may act as an indicator of embryo quality. J. Extracell. Vesicles 2, 20826 (2013).26082317

[b53] Saadeldin I. M., Kim S. J., Choi Y. B. & Lee B. C. Improvement of cloned embryos development by co-culturing with parthenotes: a possible role of exosomes/microvesicles for embryos paracrine communication. Cell Reprogram. 16, 223–234 (2014). 24773308 10.1089/cell.2014.0003 PMC4030698

[b54] Kropp J., Salih S. M. & Khatib H. Expression of microRNAs in bovine and human pre-implantation embryo culture media. Front. Genet. 5, 91 (2014). 24795753 10.3389/fgene.2014.00091 PMC4006060

[b55] Rosenbluth E. M., Shelton D. N., Wells L. M., Sparks A. E. & Van Voorhis B. J. Human embryos secrete microRNAs into culture media—a potential biomarker for implantation. Fertil. Steril. 101, 1493–1500 (2014). 24786747 10.1016/j.fertnstert.2014.01.058

[b56] Khan M. *et al.* Embryonic stem cell–derived exosomes promote endogenous repair mechanisms and enhance cardiac function following myocardial infarction. Circ. Res. 117, 52–64 (2015). 25904597 10.1161/CIRCRESAHA.117.305990 PMC4482130

[b57] Katsman D., Stackpole E. J., Domin D. R. & Farber D. B. Embryonic stem cell-derived microvesicles induce gene expression changes in Müller cells of the retina. PLoS ONE 7, e50417 (2012). 23226281 10.1371/journal.pone.0050417 PMC3511553

[b58] Aplin J. D. & Kimber S. J. Trophoblast-uterine interactions at implantation. Reprod. Biol. Endocrinol. 2, 48–48 (2004). 15236654 10.1186/1477-7827-2-48 PMC471567

[b59] Joshi A. *et al.* PARP1 during embryo implantation and its upregulation by oestradiol in mice. Reproduction 147, 765–780 (2014). 24516177 10.1530/REP-13-0588

[b60] Sun X. *et al.* Regulation of miR-101/miR-199a-3p by the epithelial sodium channel during embryo implantation: involvement of CREB phosphorylation. Reproduction 148, 559–568 (2014). 25187622 10.1530/REP-14-0386

[b61] Wang H. & Dey S. K. Roadmap to embryo implantation: clues from mouse models. Nat. Rev. Genet. 7, 185–199 (2006). 16485018 10.1038/nrg1808

[b62] Van Voorhis B. J. *In vitro* fertilization. N. Engl. J. Med. 356, 379–386 (2007). 17251534 10.1056/NEJMcp065743

[b63] Scott L. Embryological strategies for overcoming recurrent assisted reproductive technology treatment failure. Hum. Fertil. 5, 206–214 (2002). 10.1080/1464727022000199142 12477965

