# Supplementary material for: Microvesicles provide a mechanism for intercellular communication by embryonic stem cells during embryo implantation
Source: Nat Commun. 2016 Jun 15;7:11958. doi: 10.1038/ncomms11958 (PMC4912619; doi:10.1038/ncomms11958)
Supplement: Supplementary Information — Supplementary Figures 1-6, Supplementary Table 1 [file ncomms11958-s1.pdf]

Supplementary Figures and Tables

Supplementary Figures 1a and 1b

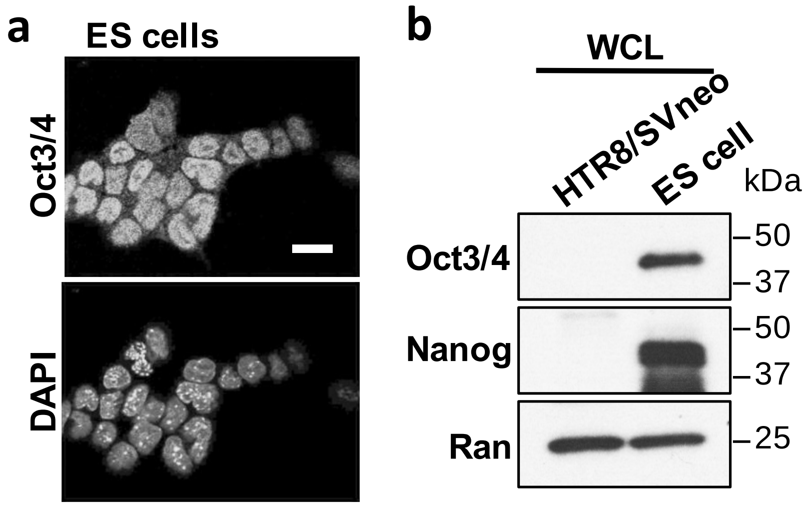

Supplementary Figure 1. Feeder layer-independent E14tg2a.4 mouse embryonic stem (ES) cells are pluripotent. **a,** Immunofluorescence image of ES cells stained with an Oct3/4 antibody, a marker of pluripotency (top). The cells were also incubated with DAPI to label nuclei (bottom). Note that all of the cells in the field are Oct3/4-positive. The scale bar is 20 μm. **b,** Whole cell lysates (WCL) of HTR8/SVneo trophoblasts and ES cells were immunoblotted for the pluripotency markers Oct3/4 and Nanog, and Ran GTPase as a loading control. Only the ES cells expressed Oct3/4 and Nanog, indicating that they are indeed pluripotent.

Supplementary Figures 2a-2f

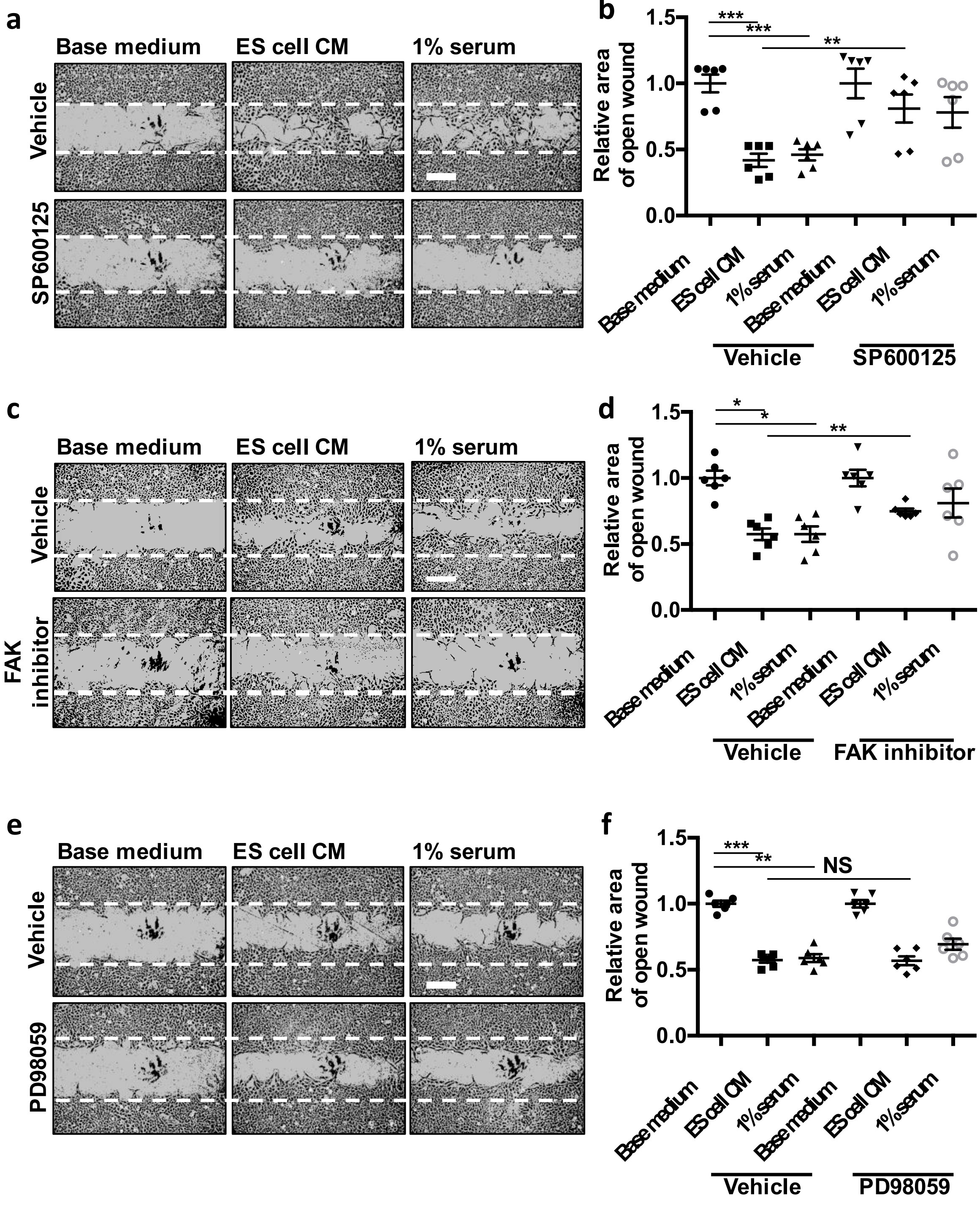

Supplementary Figure 2. FAK and JNK activation are important for HTR8/SVneo trophoblasts to migrate in response to treatment with ES cell CM. **a**-**f**, Wound closure assays were performed on HTR8/SVneo cells cultured in ES cell base medium lacking serum and LIF (Base medium), the CM collected from ES cells cultured in the same medium (ES cell CM), or medium containing 1% serum. Each culturing condition was treated further with either DMSO (Vehicle) or (**a**) SP600125, (**c**) FAK inhibitor III (FAK inhibitor) or (**e**) PD98059. Images of the cells are shown. The dashed line indicates the width of the original wound. The scale bar is 250 μm. The assays in (**a, c**, **e**) were quantified and plotted in (**b, d**, **f**) as the relative area of open wound. All values shown are presented as mean ± s.e.m. (n ≥ 3 independent experiments for each assay). Differences were analyzed using Student's t-test; *, P<0.05, **, P<0.01, ***, P<0.001, NS, not significant.

Supplementary Figures 3a-g

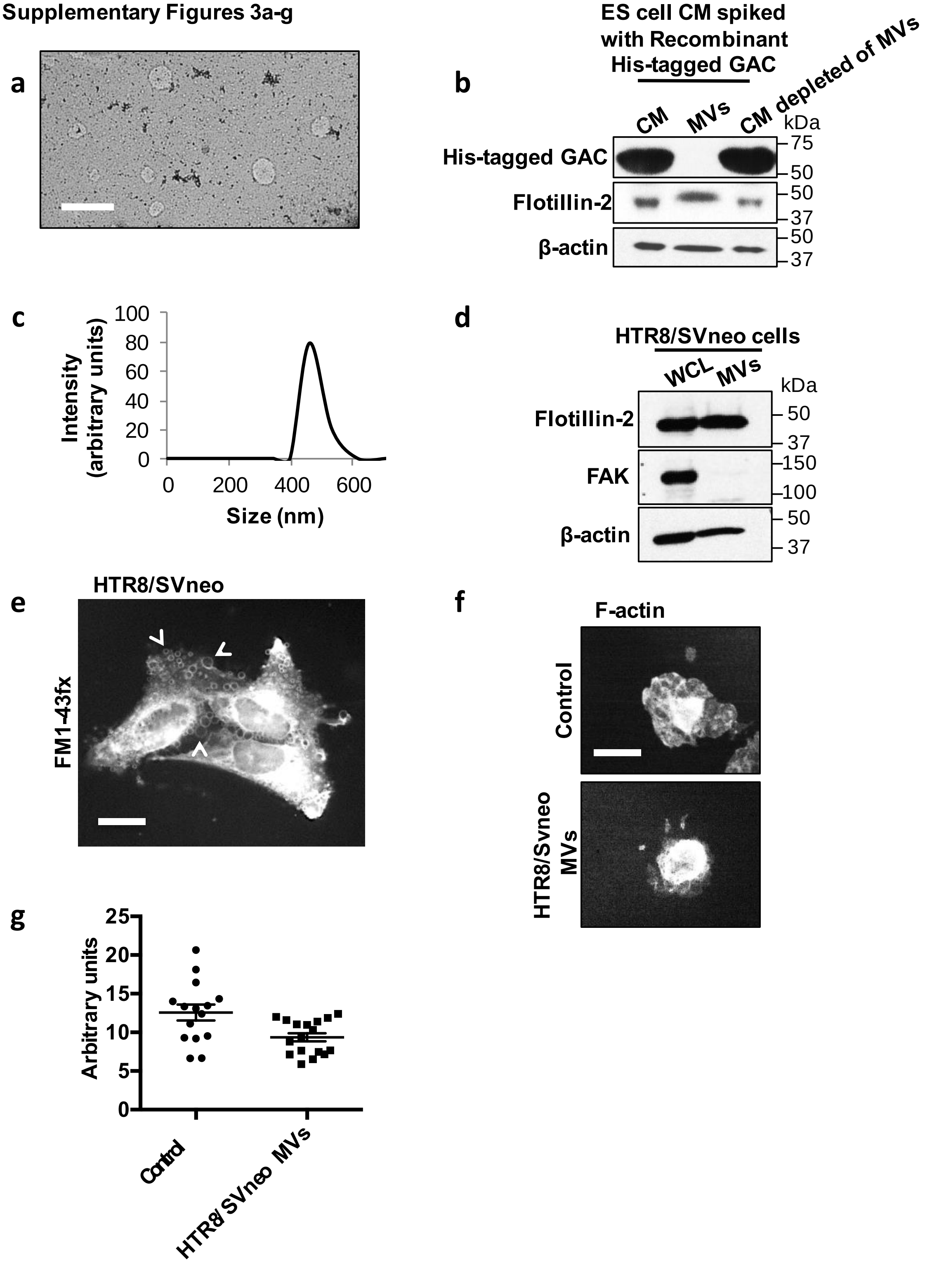

Supplementary Figure 3. ES cells and trophoblasts generate MVs. **a**, Transmission electron microscopy (TEM) image of MVs isolated from ES cells. Scale bar is 1 μm. **b**, To show that the MV preparations are free of soluble proteins, 40 ml of CM from ES cells was supplemented with 20 μg of a tagged recombinant form of Glutaminase C (His-tagged GAC) that has a molecular weight of ~65 kDa. The medium was clarified of intact cells and cell debris and divided into two equal parts. One part (20 ml) was concentrated to 400 μl using a centrifugal filter with a nominal molecular weight limit of 10 kDa. The rest was filtered with a 0.22 μm filter and then rinsed extensively with PBS. The MVs retained by the filter were lysed in 400 μl of mammalian lysis buffer, while the flow-through was concentrated to 400 μl as well. All of the samples were immunoblotted for His-tagged GAC, the MV marker flotillin-2, and β-actin as a loading control. Note that His-tagged GAC was detected in the CM (first lane) and the flow-through (third lane), but not in the MVs (second lane), demonstrating that the MV isolation procedure efficiently removes soluble proteins. **c,** DLS analysis performed on MVs isolated from trophoblasts show that they ranged in size from ~400-600 nm. **d,** Lysates of HTR8/SVneo trophoblasts (WCL) and their MVs were immunoblotted for the MV marker flotillin-2, the cytosolic marker FAK, and β-actin as a loading control. **e,** Fluorescence microscopy image of trophoblasts stained with the membrane dye FM1-43fx. Some of the MVs decorating the surfaces of the cells are denoted with arrowheads. Scale bar is 20 μm. **f**, E3.5 blastocysts were harvested and placed in dishes containing blastocyst culturing medium supplemented without (Control) or with HTR8/SVneo trophoblast MVs. Two days later, the blastocysts were stained with rhodamine-conjugated phalloidin and visualized by fluorescence microscopy. Images of the blastocysts are shown. The scale bar is 100 μm. **g**, The assays in (**f**) were quantified and graphed as relative area of

blastocyst outgrowth. All values shown are presented as mean ± s.e.m. (n = 3 independent experiments for each assay).

Supplementary Figures 4a-e

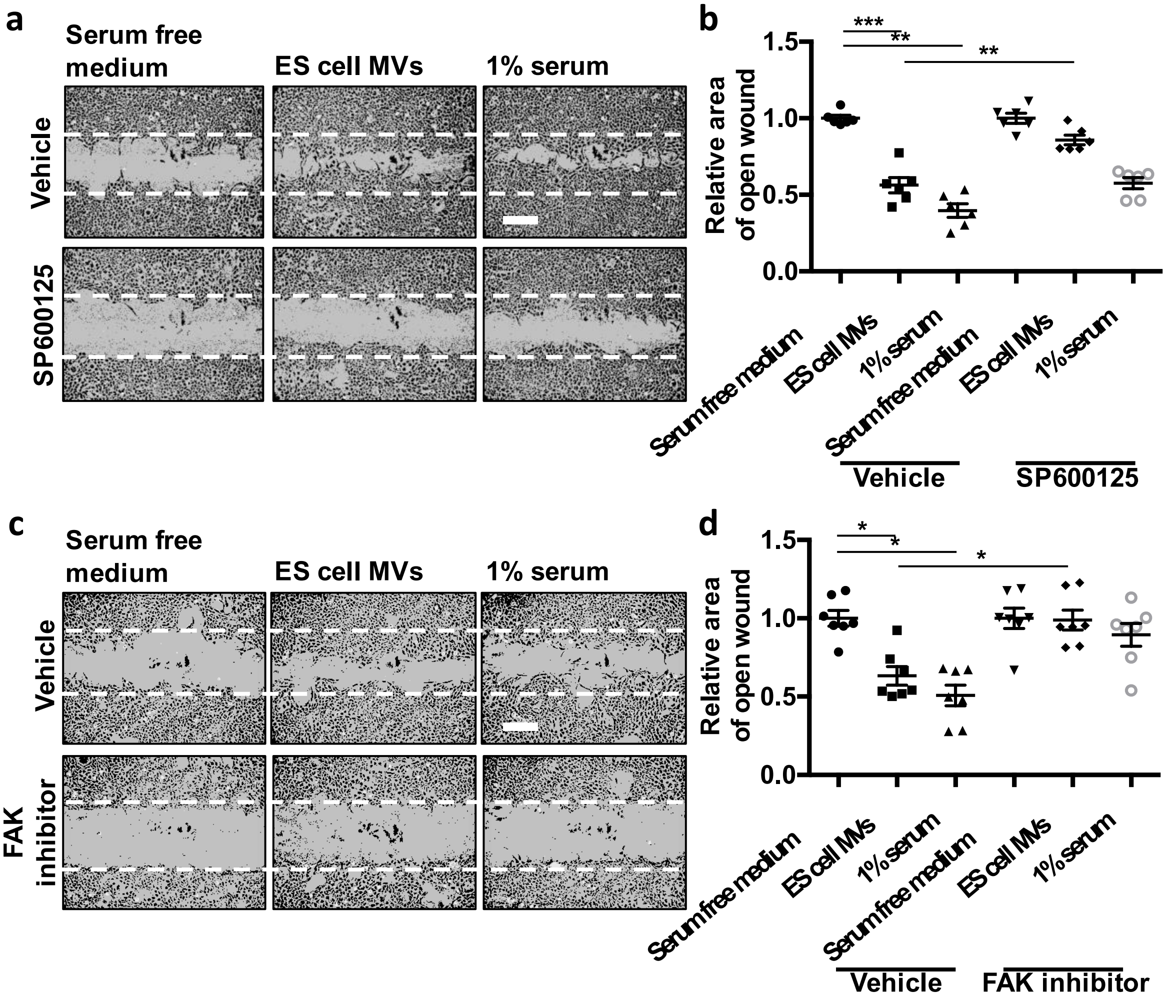

e

Blastocyst outgrowth assay

Treatment	Blastocyst attachment	Blastocyst outgrowth
Control (untreated)	9/9	9/9
FAK inhibitor	1/9	0/9
JNK inhibitor	0/8	0/8

Supplementary Figure 4. FAK and JNK activation are important for HTR8/SVneo cells to migrate in response to treatment with ES cell MVs. **a-d**, Wound closure assays were performed on HTR8/SVneo cells cultured in serum free medium supplemented without (Serum free medium) or with either MVs from ES cells (ES cell MVs), or medium containing 1% serum. Each culturing condition was treated further with either DMSO (Vehicle) or (**a**) SP600125 or (**c**) FAK inhibitor III (FAK inhibitor). Images of the cells are shown. The dashed line indicates the width of the original wound. The scale bar is 250 μm. The assays in (**a, c**) were quantified and plotted in (**b, d**) as the relative area of open wound. **e**, E3.5 blastocysts were harvested and placed in dishes containing blastocyst culturing medium supplemented without (Control) or with either FAK inhibitor III (FAK inhibitor) or SP600125 (JNK inhibitor). Two days later, the number of blastocysts that attached and formed outgrowths onto the dish was determined. All values shown are presented as mean ± s.e.m. (n ≥ 3 independent experiments for each assay). Differences were analyzed using Student's t-test; *, P<0.05, **, P<0.01, ***, P<0.001.

Supplementary Figures 5a-f

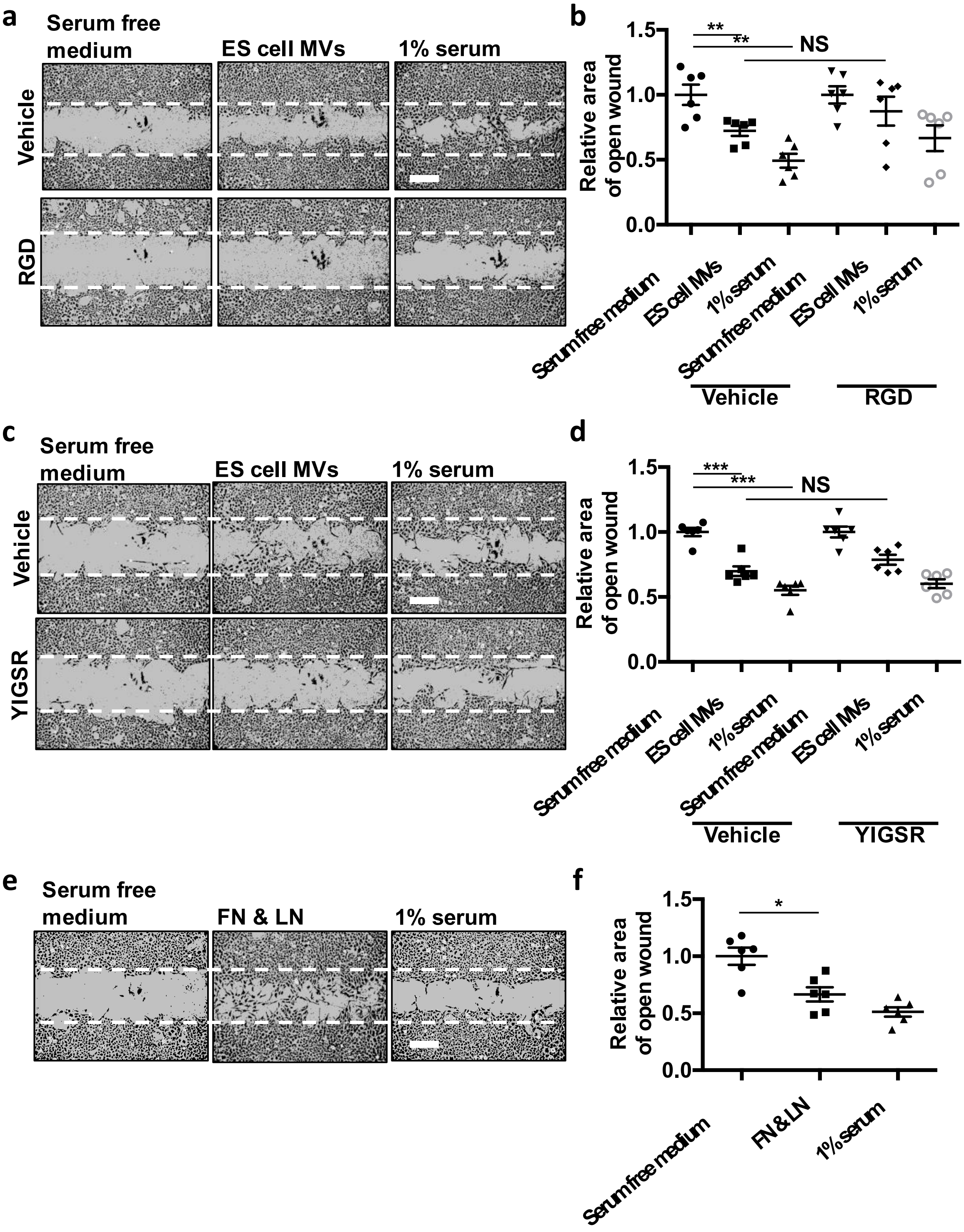

Supplementary Figure 5. Inhibiting the MV-associated fibronectin or laminin from binding their corresponding receptors expressed on trophoblasts does not interfere with their ability to migrate. **a**, Wound closure assays were performed on HTR8/SVneo cells cultured in serum free medium supplemented without (Serum free medium) or with either MVs from ES cells (ES cell MVs) or 1% serum. Each culturing condition was treated further with vehicle (top panels) or the RGD inhibitory peptide (bottom panels). Images of the cells maintained under each condition are shown. The dashed line indicates the width of the original wound. The scale bar is 250 μm. **b**, The assays in (**a**) were quantified and plotted as the relative area of open wound. **c**, Wound closure assays were performed as in (**a**), except that HTR8/SVneo cells were treated with the YIGSR inhibitory peptide (bottom panels). **d**, The assays in (**c**) were quantified and plotted as the relative area of open wound. **e**, Wound closure assays were performed on HTR8/SVneo cells cultured in serum free medium supplemented without (Serum free medium) or with either purified forms of fibronectin and laminin (FN & LN) or 1% serum. Images of the cells maintained under each condition are shown. The dashed line indicates the width of the original wound. The scale bar is 250 μm. **f**, The assays in (**e**) were quantified and plotted as the relative area of open wound. All values shown are presented as mean ± s.e.m. ($n \geq 3$ independent experiments for each assay), Differences were analyzed using Student's t-test; *, $P<0.05$, **, $P<0.01$, ***, $P<0.001$, NS, not significant.

Supplementary Figure 6

<u>Figure 1a</u>

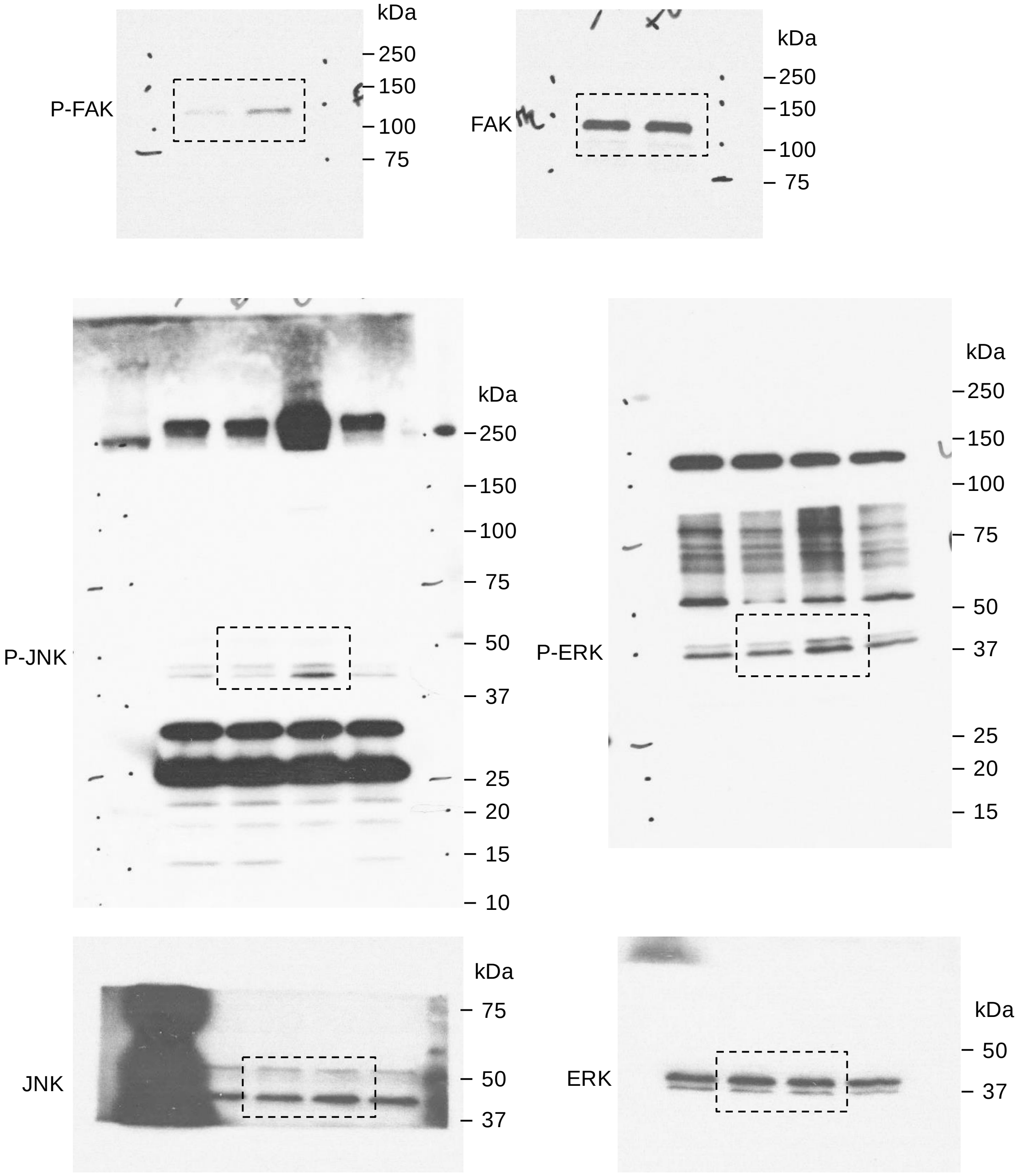

Supplementary Fig. 6 continued

Figure 1i

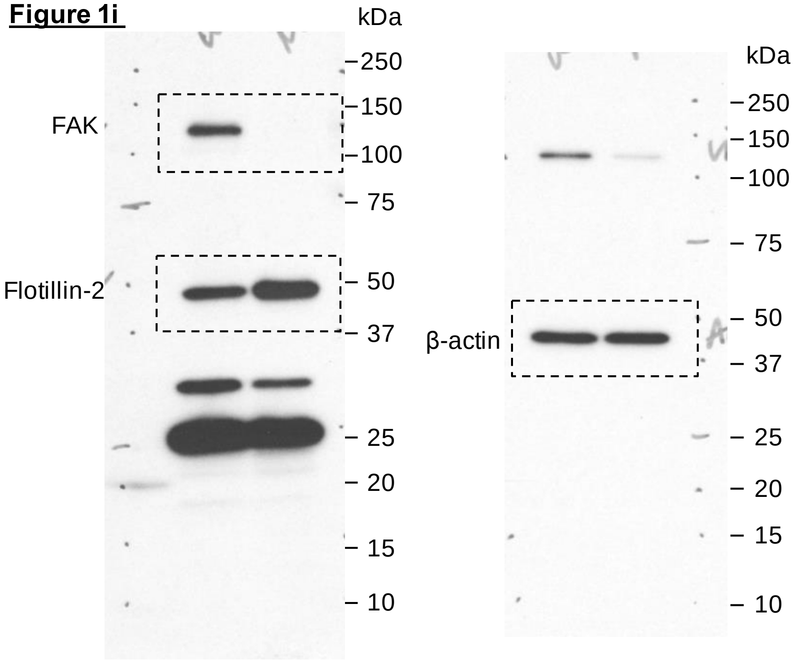

Figure 2a

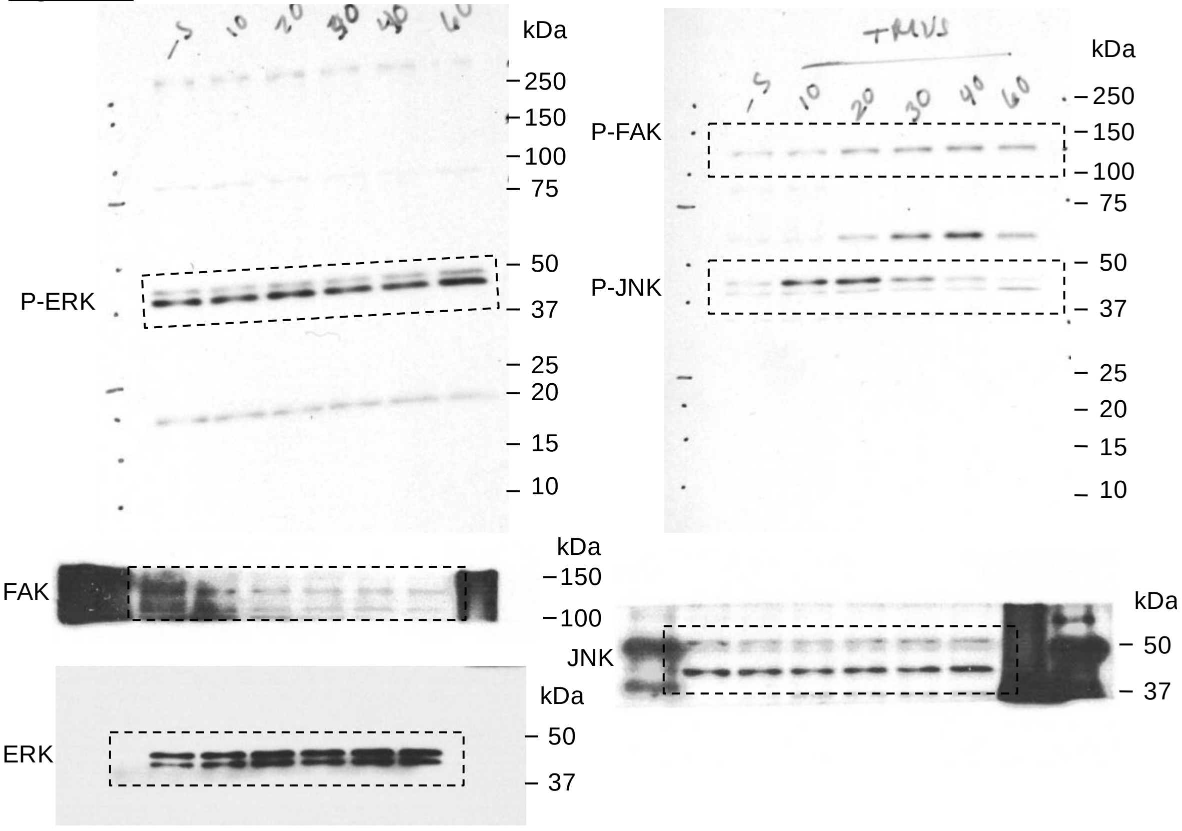

Supplementary Figure 6 continued

Figure 3b

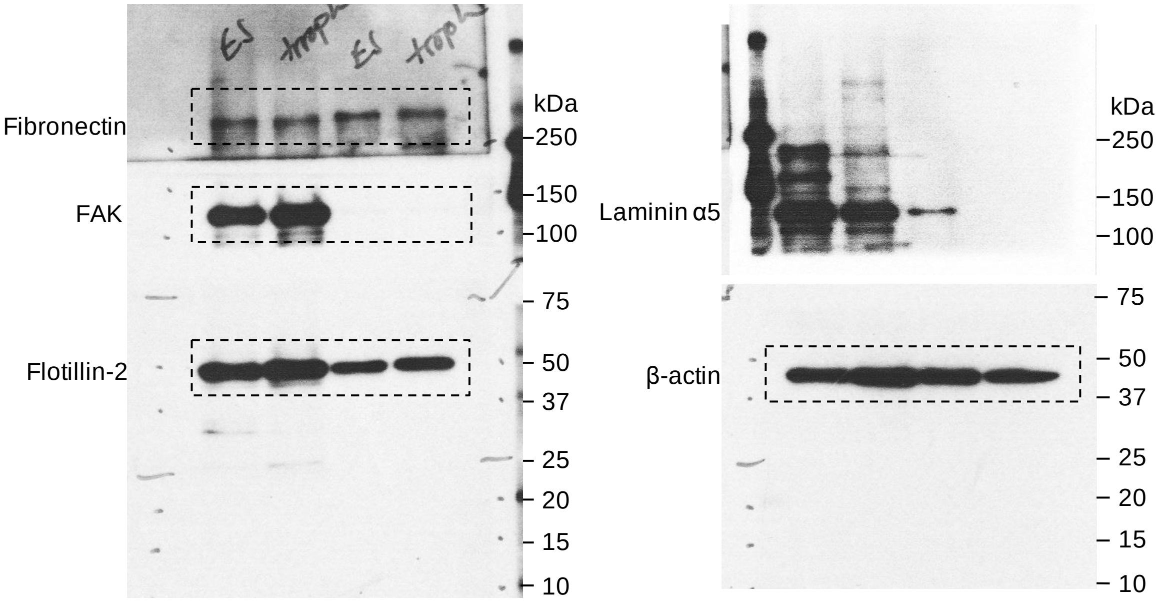

Figure 3c

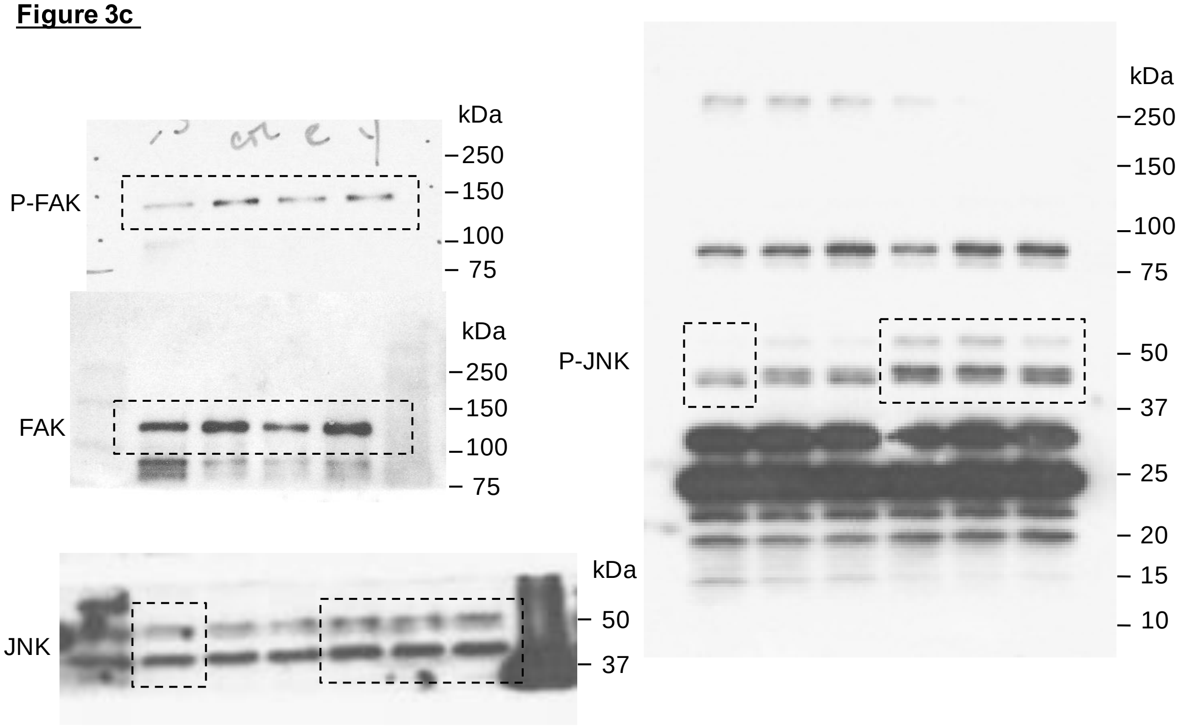

Supplementary Figure 6 continued

Figure 3d

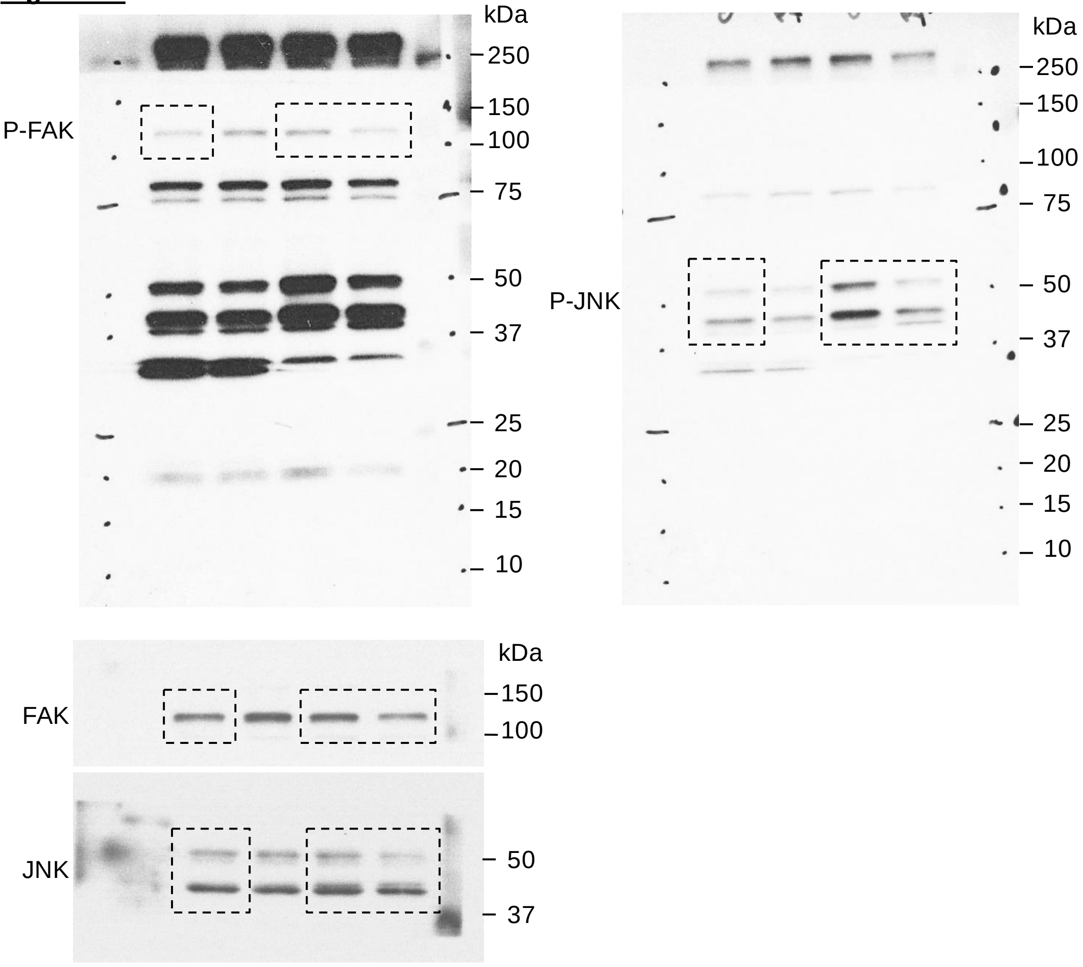

Supplementary Figure 6 continued

Figure 4b

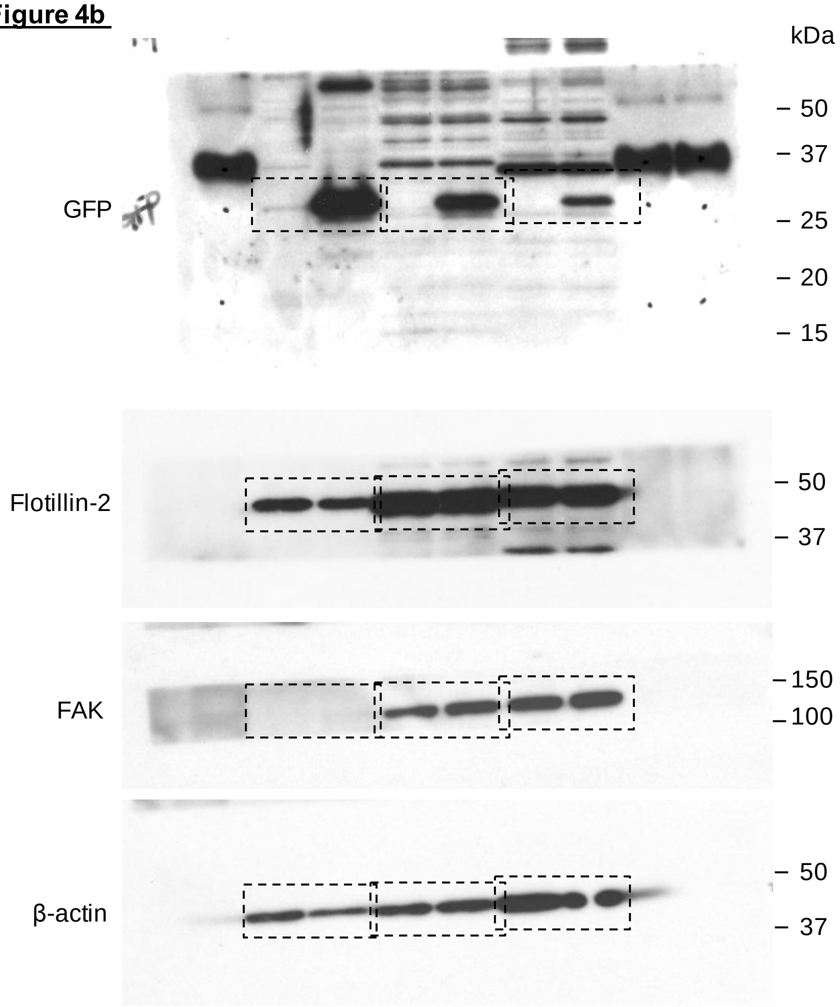

Supplementary Figure 6 continued

SI Figure 1b

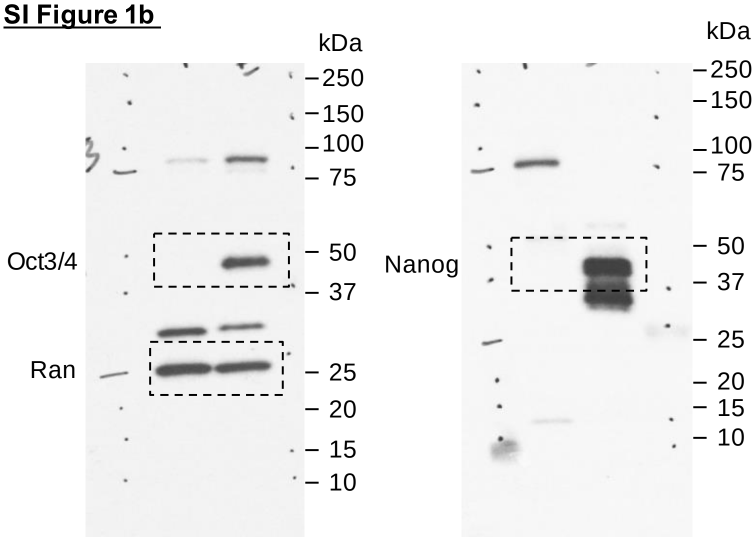

SI Figure 3b

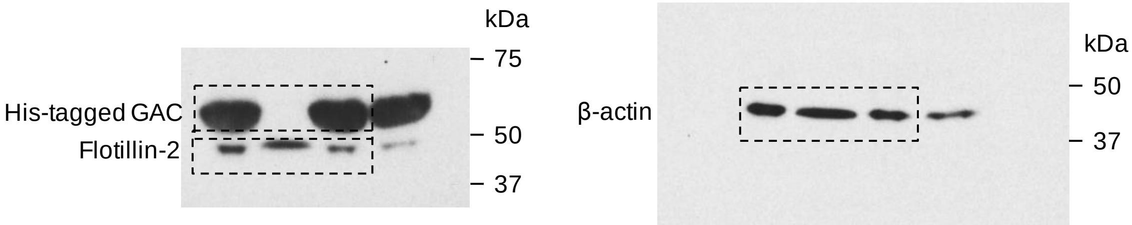

SI Figure 3d

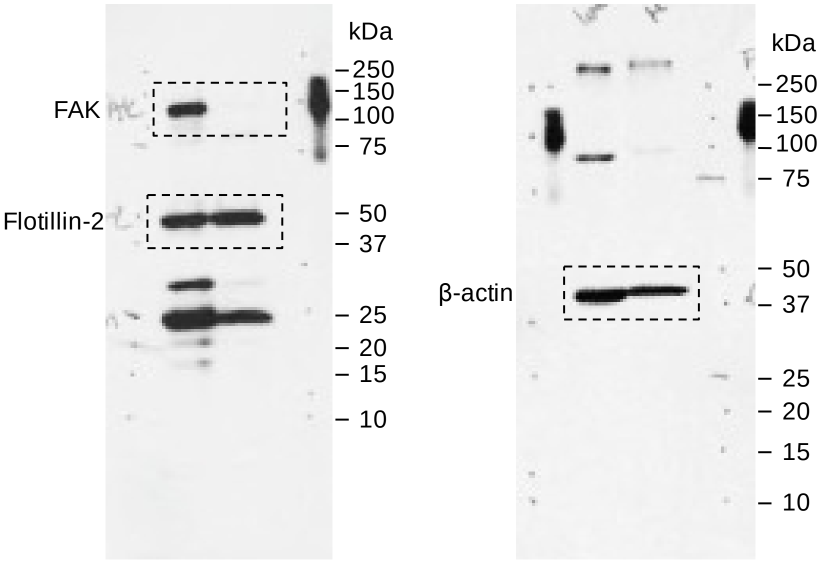

Supplementary Figure 6. Full, uncropped images of all immunoblots shown in the main figures, as well as supplementary figures. Molecular weight markers (Bio-Rad Precision Plus Dual Color Protein Standards) are indicated on the right of all blots. Note that on some blots, a second molecular weight marker (Bio-Rad Kaleidoscope Pre-stained Standards) was also used. However, only the sizes for the Bio-Rad Precision Plus Dual Color Protein Standards are indicated.

Supplementary Table 1. Raw data and calculations for the Wilcoxon signed-rank test performed on the results shown in Fig. 4f.[1]

1	2	3	4	5	6	7	8
Female mouse ID#	Total # of blastocysts transferred per uterine horn	# of blastocysts injected with MVs that implanted in the right uterine horn	# of blastocysts injected with vehicle that implanted in the left uterine horn	Difference between columns 3 and 4	Rank of the absolute value of the differences in column 5	Rank values from column 6 with a positive # in column 5	Rank values from column 6 with a negative # in column 5
1	7	5	6	-1	2	-	2
2	7	7	5	2	6	6	-
3	8	7	4	3	9	9	-
4	8	7	5	2	6	6	-
5	8	6	7	-1	2	-	2
6	7	7	3	4	10.5	10.5	-
7	7	3	5	-2	6	-	6
8	5	4	0	4	10.5	10.5	-
9	6	4	4	0	0	0	-
10	7	5	4	1	2	2	-
11	7	7	5	2	6	6	-
12	7	7	5	2	6	6	-
					**Sum**	**56**	**10**

W value=10
n=11
p=0.05

Raw data and calculations for the Wilcoxon signed-rank test performed on the results shown in Fig. 4f. Column 1: the pseudopregnant mice were labeled with an identifier. Column 2: lists the number of blastocysts injected with either vehicle alone or ES cell MVs and then transferred to the left (blastocysts injected with vehicle) or right (blastocysts injected with MVs) uterine horn in a given mouse. Columns 3 and 4: the number of blastocysts that implanted on either the right (column 3) or left (column 4) uterine horn of a given mouse. A Wilcoxon signed-rank analysis was then performed (see columns 5 through 8) using the information in columns 3 and 4. Column 5: the difference between the number of embryos that implanted in the right (column 3) and the left (column 4) uterine horns of a given mouse. Note that mouse number 9 was excluded from further calculations since implantation rates were equal for each condition (n=11). Column 6: the absolute values of the differences in column 5 were assigned ranks, from 1 to 11, from lowest (1) to highest (11). In the case of a tie, the mean rank was assigned to multiple differences. Columns 7 and 8: the ranks in column 6 were then sorted into two columns based on whether their corresponding difference in column 5 was positive (column 7) or negative (column 8). The sorted rank values were then summed. The sum of the negative rank values (column 8, bottom), representing the instances where blastocysts injected with vehicle implanted more efficiently than those injected with MVs, represents the “W value.” By assessing a chart of W values, for n=11, the W value must be 10 or less to achieve statistical significance of p=0.05 (two-tailed test). Therefore, these data indicate that there is a statistically significant difference between the implantation rates of the blastocysts injected with vehicle and those injected with MVs.[1]
