# Supplementary material for: Microvesicles provide a mechanism for intercellular communication by embryonic stem cells during embryo implantation
Source: Nat Commun. 2016 Jun 15;7:11958. doi: 10.1038/ncomms11958 (PMC4912619; doi:10.1038/ncomms11958)
Supplement: Peer Review File — Proteins identified in MVs isolated from ES cells using mass spectrometry [file ncomms11958-s3.pdf]

## Editorial Note

This manuscript has been previously reviewed at another journal that is not operating a transparent peer review scheme. This document only contains reviewer comments and rebuttal letters for versions considered at Nature Communications.

**Reviewers' Comments**

**Reviewer #1 (Remarks to the Author)**

I had reviewed this article by Desrochers et al for another Nature journal, and I can see that the authors have done a good job at including many corrections in this revised manuscript, to answer my previous comments. In particular, concerns about number of experiments performed and reproducibility of the experiments are now addressed by proper explanation of the experimental set ups, and representations of results as dot plots instead of histograms. In addition, detection of the total non-phosphorylated versions of the signalling proteins analysed by WB and relative quantifications are now provided, although only apparently for one experiment each time. Finally, the authors have now included a control of trophoblast-derived MVs in the wound-healing assays, showing absence of effect, as opposed to a wound-closure effect of ES-derived MVs. They have also performed additional controls to answer the other reviewers comments, properly ruling out the involvement of soluble factors from the conditioned medium in the functions observed. The authors can be thanked for including all these corrections and controls.

Concerning my previous comment on the unclear relevance of these observations to an in vivo situation, where spontaneous secretion of MVs by ES cells should be demonstrated to be important for embryo implantation, I can accept the authors response, that they have not managed to find any tools to inhibit specifically MV secretion by these cells, and thus could not answer this concern.

Thus, convincingly proving that ES-MVs have a functional relevance in the mixed in vitro/in vivo setting of MVs injection in blastocystes seems now the only remaining requirement, for this article to be sufficiently novel to qualify for Nature communications. Indeed, as mentioned in my previous review, effects of MVs or EVs in wound healing assays are not really novel, even if they had not been shown before for ES cell-derived EVs and recipient trophoblast cells. Unfortunately, this last part of the article remains insufficiently convincing (figures 4-5), because the authors have not used here the relevant control of trophoblast-derived MVs in the in vivo assays. Again, since the model is of a specific ES-to-trophoblast communication by MVs, trophoblast-to-trophoblast communication by MVs in the blastocyst setting must be excluded. This would involve incubating trophoblast cells with trophoblast-derived PM-GFP+ or FM1-43fx+ MVs and showing no or low capture as compared to ES-MVs (fig 4b,c), injecting blastocystes with PM-GFP+ trophoblast-derived MVs and showing absence of binding to trophoblast in several independent embryos (or even possibly binding instead to the inner cell mass) (fig 4d,e), and finally injecting trophoblast-derived MVs in embryos, and monitoring implantation (fig4f). This latter experiment is very heavy and time-consuming, and may appear difficult to perform, but given the now obvious variability of effect of ES-MVs, and border-line statistical significance of the MV effect in this assay, I am not convinced of this effect in absence of a proper control. Careful analysis of the data indeed shows increase of the mean implantation by MV injection, but this increase is observed in 7 mice, whereas decrease or no effect is observed in 4 mice (1 experiment with no counterpart control). My calculation of the paired-non parametric Wilcoxon signed-rank test based on the values observed on fig4f gives a p-value of 0.113 (thus not $<0.05$), whereas a parametric student t-test comparing the

means gives a p-value of 0.0491. The authors specify the use of Wilcoxon signed-rank test for this particular experiment (M&M), thus should not indicate * here.

If the editors consider that strong significance of the blastocyst implantation experiments (fig4f) is not necessary for the message of the paper and can be overlooked, at least controls showing absence of association (or not) of trophoblast MVs to trophoblast cells, in vitro (4c) and in vivo after injection in blastocyst (4d-e) should be provided. Note that absence of GFP fluorescence in trophectoderm after injection of non-fluorescent EVs (fig4e) is expected, and does not represent a negative control.
In addition, the WB chara
cterizing trophoblast-MVs (suppl figure 3d) should been shown in main figure 3b, side-by-side with ES-MVs, and including staining for fibronectin and laminin-5 : absence of these two proteins in trophoblast-MVs is expected, and if these proteins are present, the authors must re-consider their model of ES-EV-associated LN and FN being responsible for the wound-healing effect.

**Minor comments:**
The control showing no wound-healing effect of trophoblast-MVs in vitro (figure Suppl 3g) must be part of main figure 2. Label « GFP » in figure 4c where FM1-43fx-labeled MVs have been used seems inappropriate amount of materials loaded in the WB is not indicated position of the MW markers in WB is not indicated: which band for eg P-JNK is considered specific and used for the quantification line 74 : « a significant percentage of the ES cell population » is not appropriate. Since 60% of the cells are labelled, it could be changed for « a majority of the ES cells »

**Reviewer #2 (Remarks to the Author)**

The figures have been greatly improved by the revision. Some of the key experimental details are still hard to find in the text. For example, information about how the conditioned medium was collected from the cells (p. 2) could be more explicit, as could the details of filtering the microvesicle prep. At the very least, the information provided in the rebuttal for the reviewers should be included in the Methods section, but it would be even better if the manuscript text and figure legends more clearly explained that the base medium does not contain serum, that the conditioned medium did not contain serum, etc. As is, the figures still seem like the controls and experiments are apples and oranges.

**Reviewer #3 (Remarks to the Author)**

The authors have made a big effort to address all my previous concerns and improved the manuscript significantly. I am satisfied with the revised product.

**Point-By-Point**

**Reviewer #1**

**Major comments:**

*I had reviewed this article by Desrochers et al for another Nature journal, and I can see that the authors have done a good job at including many corrections in this revised manuscript, to answer my previous comments. In particular, concerns about number of experiments performed and reproducibility of the experiments are now addressed by proper explanation of the experimental set ups, and representations of results as dot plots instead of histograms. In addition, detection of the total non-phosphorylated versions of the signalling proteins analysed by WB and relative quantifications are now provided.*

*Finally, the authors have now included a control of trophoblast-derived MVs in the wound-healing assays, showing absence of effect, as opposed to a wound-closure effect of ES-derived MVs. They have also performed additional controls to answer the other reviewers' comments, properly ruling out the involvement of soluble factors from the conditioned medium in the functions observed. The authors can be thanked for including all these corrections and controls.*

*Concerning my previous comment on the unclear relevance of these observations to an in vivo situation, where spontaneous secretion of MVs by ES cells should be demonstrated to be important for embryo implantation, I can accept the authors response, that they have not managed to find any tools to inhibit specifically MV secretion by these cells, and thus could not answer this concern. Thus, convincingly proving that ES-MVs have a functional relevance in the mixed in vitro/in vivo setting of MVs injection in blastocysts seems now the only remaining requirement for this article to be sufficiently novel to qualify for Nature Communications. Indeed, as mentioned in my previous review, effects of MVs or EVs in wound healing assays are not really novel, even if they had not been shown before for ES cell-derived EVs and recipient trophoblast cells. Unfortunately, this last part of the article remains insufficiently convincing (figures 4-5), because the authors have not used here the relevant control of trophoblastderived*

*MVs in the in vivo assays.*

*Again, since the model is of a specific ES-to-trophoblast communication by MVs, trophoblast-to-trophoblast communication by MVs in the blastocyst setting must be excluded. This would involve incubating trophoblast cells with trophoblast-derived PM-GFP+ or FM1-43fx+ MVs and showing no or low capture as compared to ES-MVs (fig 4b,c), injecting blastocysts with PM-GFP+ trophoblast-derived MVs and showing absence of binding to trophoblast in several independent embryos (or even possibly binding instead to the inner cell mass) (fig 4d,e), and finally injecting trophoblast-derived MVs in embryos, and monitoring implantation (fig4f). This latter experiment is very heavy and time-consuming, and may appear difficult to perform, but given the now obvious variability of effect of ES-MVs, and border-line statistical significance of the MV effect in this assay, I am not convinced of this effect in absence of a proper control. Careful analysis of the data indeed shows increase of the mean implantation by MV injection, but this increase is observed in 7 mice, whereas decrease or no effect is observed in 4 mice (1 experiment with no counterpart control). My calculation of the paired-non parametric Wilcoxon signed-rank test based on the values observed on*

*fig4f gives a p-value of 0.113 (thus not <0.05), whereas a parametric student t-test comparing the means gives a p-value of 0.0491. The authors specify the use of Wilcoxon signed-rank test for this particular experiment (M&M), thus should not indicate * here.*

*If the editors consider that strong significance of the blastocyst implantation experiments (fig4f) is not necessary for the message of the paper and can be overlooked, at least controls showing absence of association (or not) of trophoblast MVs to trophoblast cells, in vitro (4c) and in vivo after injection in blastocyst (4d-e) should be provided. Note that absence of GFP fluorescence in trophectoderm after injection of non-fluorescent EVs (fig4e) is expected, and does not represent a negative control. In addition, the WB characterizing trophoblast-MVs (suppl figure 3d) should been shown in main figure 3b, side-by-side with ES-MVs, and including staining for fibronectin and laminin-5: absence of these two proteins in trophoblast-MVs is expected, and if these proteins are present, the authors must reconsider their model of ES-EV-associated LN and FN being responsible for the wound-healing effect.*

We have addressed each of the reviewer's comments/questions individually as follows:

**1)** *This last part of the article remains insufficiently convincing (figures 4-5), because the authors have not used the relevant control of trophoblast-derived MVs in the in vivo assays. Again, since the model is of a specific ES-to-trophoblast communication by MVs, trophoblast-to-trophoblast communication by MVs in the blastocyst setting must be excluded. This would involve incubating trophoblast cells with trophoblast-derived PM-GFP+ or FM1-43fx+ MVs and showing no or low capture as compared to ESMVs (figs 4b, 4c), injecting blastocysts with PM-GFP+ trophoblast-derived MVs and showing absence of binding to trophoblast in several independent embryos (or even possibly binding instead to the inner cell mass) (fig 4d,e), and finally injecting trophoblast-derived MVs in embryos, and monitoring implantation (fig4f). This latter experiment is very heavy and time-consuming, and may appear difficult to perform, but given the now obvious variability of effect of ES-MVs, and border-line statistical significance of the MV effect in this assay, I am not convinced of this effect in absence of a proper control.*

We injected blastocysts (in 3 separate experiments for a total of 20 blastocysts) with MVs derived from PM-GFP-expressing trophoblasts and then determined whether they could be transferred to the trophectoderm layer, similar to embryonic stem (ES) cell MVs. The results of this experiment showed that only 1/20 blastocysts had detectable levels of GFP signal along their trophectoderm. Instead, GFP signal was more often (i.e. in 7/20 blastocysts) concentrated in the inner cell mass (ICM), suggesting that trophoblast MVs are not efficiently taken-up by other trophoblasts. These findings have been included in Figures 4d and 4e, and are described on page 12 of the revised manuscript.

As pointed out by the reviewer, performing implantation experiments using blastocysts injected with MVs from trophoblast are extremely time-consuming and difficult to perform (in fact, the results shown in Figure 4f took us ~5 months to generate). However, we also appreciate that finding additional ways to show that trophoblast MVs are not able to function like ES cell MVs and promote embryo implantation would help further help to support our claims. Thus, we determined how treating isolated E3.5 blastocysts with MVs from trophoblasts affected their ability to attach to a

substrate and migrate; i.e. we performed blastocyst outgrowth assays, which are frequently used to study the initial steps of the implantation process. We found that, while the ES cell MVs increased blastocyst outgrowths by about 3-fold, compared to their untreated control counterparts (see Figures 2f and 2g), the trophoblasts MVs failed to have the same effect. These findings are now shown in the newly added Supplementary Figures 3f and 3g, and are described starting with the first full paragraph on page 8 of the revised manuscript.

***2)*** *…given the now obvious variability of effect of ES-MVs, and border-line statistical significance of the MV effect in this assay, I am not convinced of this effect in absence of a proper control. Careful analysis of the data indeed shows increase of the mean implantation by MV injection, but this increase is observed in 7 mice, whereas decrease or no effect is observed in 4 mice (1 experiment with no counterpart control). My calculation of the paired-non parametric Wilcoxon signed-rank test based on the values observed on fig4f gives a p-value of 0.113 (thus not <0.05), whereas a parametric student test comparing the means gives a p-value of 0.0491. The authors specify the use of Wilcoxon signedrank test for this particular experiment (M&M), thus should not indicate * here.*

We recognize that it is difficult for the reviewer to accurately carry-out a Wilcoxon signed-rank test on the results shown in Figure 4f without access to all the experimental data. Thus, we have now added Supplementary Figure 6, which provides a table containing the raw data, as well as many of the calculations used to determine the differences and ranks that were used for this analysis, to the revised manuscript. In the corresponding legend for this figure, we have also included detailed, step-by-step instructions for how the results for the Wilcoxon signed-rank test were determined.

Regarding the apparent discrepancy between Reviewer #1's p-value calculation (p=0.113) and our own (p≤0.05), we think it is most likely due to the reviewer's assumption that the number of blastocysts that were injected into each uterine horn of a pseudo-pregnant mouse was always 7 (as would be deduced by dividing 84 vehicle- or MV-injected blastocysts by 12 mice). However, this would be incorrect. Rather, due to differences in the number of blastocysts that were harvested for a given experiment, the exact number of blastocysts that were transferred to surrogate mice varied from 5-8 (although it is important to note that the number of blastocysts injected into each uterine horn of a given mouse was always the same). If the reviewer again performs the Wilcoxon signed-rank test taking this new information into account (see Supplementary Figure 6), we feel confident that he/she will get a statistically significant difference in the implantation rates of blastocysts injected with vehicle-alone and those injected with ES cell MVs (i.e. p≤0.05).

***3)*** *If the editors consider that strong significance of the blastocyst implantation experiments (fig. 4f) is not necessary for the message of the paper and can be overlooked, at least controls showing absence of association (or not) of trophoblast MVs to trophoblast cells, in vitro (4c) and in vivo after injection in blastocyst (4d-e) should be provided. Note that absence of GFP fluorescence in trophectoderm after injection of non-fluorescent EVs (fig4e) is expected, and does not represent a negative control.*

This point has already been addressed in our response to Major Comment #1 (above).

***4)** In addition, the Western blot characterizing trophoblast-MVs (Supplementary Figure 3d) should been shown in main figure 3b, side-by-side with ES-MVs, and include staining for fibronectin and laminin-5.*
*Absence of these two proteins in trophoblast-MVs is expected, and if these proteins are present, the authors must re-consider their model of ES-EV-associated LN and FN being responsible for the woundhealing-effect.*

Western blot analyses using fibronectin and laminin α5 antibodies were carried-out on equivalent amounts (5.0 μg) of MV lysates derived from either ES cells or trophoblasts run side-by-side on the same gel. The resulting blots showed that fibronectin expression levels were similar in these MVs, however the levels of laminin α5 were very different. Specifically, laminin α5 was only detected in the ES cell MVs and not in MVs isolated from trophoblasts. It is also worth emphasizing that this experiment was performed on three separate occasions using different MV preparations, each time yielding similar results. The new immunoblot showing the relative expression levels of fibronectin and laminin α5 in MVs from ES cells and trophoblasts now replaces the original immunoblot shown in Figure 3b.

The fact that trophoblast MVs do not contain laminin α5 (see Figure 3b), are unable to be efficiently transferred to the trophectoderm layer when injected into blastocysts (see Figures 4d and 4e), do not enhance blastocyst outgrowths (see Supplementary Figures 3f and 3g), and do not promote the migration of trophoblasts in wound-healing assays (see Figures 2h and 2i), all point to the idea that trophoblast MVs cannot recapitulate the effects of ES cell MVs. We believe that the addition of all of this new information to the revised manuscript has significantly strengthened it. Moreover, these findings lend further support to our findings that MVs generated by ES cells are uniquely capable of eliciting the migratory phenotype in trophoblasts that underlies embryo implantation.

**Minor comments:**
***1)** The control showing no wound-healing effect of trophoblast-MVs in vitro (figure Suppl 3g) must be part of main figure 2.*

These results have now been moved to Figure 2 (see Figure 2h and 2i).

***2)** Label « GFP » in figure 4c where FM1-43fx-labeled MVs have been used seems inappropriate.*
The label in question has now been changed to *FM1-43fx*.

***3)** Amount of materials loaded in the WB is not indicated.*
For the Western blots performed on MVs derived from ES cells and/or trophoblasts, 5.0 μg of each MV lysate was used. However, for all of the other experiments involving Western blots, including probing ES cell lysates for markers of pluripotency (Supplementary Figure 1b) and examining the ability of MVs or conditioned medium to stimulate signalling activities in trophoblasts, 15 μg of each lysate was used.
This information has now been added to Material and Methods under the subheading *Immunoblot analysis*.

***4)*** *Position of the MW markers in WB is not indicated: which band for e.g. P-JNK is considered specific and used for the quantification?*

Molecular weight markers have been added to all immunoblots (see Figures 1a, 1i, 2a, 3b, 3c, 3d, 4b, and Supplementary Figures 1b, 3b and 3d), and, for consistency, scale bars have been included on all microscopy images (see Figures 1b, 1g, 2b, 2d, 2f, 2h, 3e, 4c, 4d, and Supplementary Figures 1a, 2a, 2c, 2e, 3a, 3e, 3f, 4a, 4c, 5a, 5c, and 5e). Moreover, a figure has been added to the revised manuscript (Supplementary Figure 7) that contains all of the original, uncropped images of the immunoblots used to generate the figures in the manuscript.

Regarding which of the two bands detected using antibodies that recognize either activated (phosphorylated) forms of JNK (P-JNK) or ERK (P-ERK) were used for quantification, the answer is both were used. This has now been explicitly stated in Materials and Methods under the subheading *Immunoblot analysis*.

***5)*** *Line 74: « a significant percentage of the ES cell population » is not appropriate. Since 60% of the cells are labelled, it could be changed to « a majority of the ES cells »*

This sentence has now been modified accordingly.

**Reviewer #2**

*The figures have been greatly improved by the revision.*

*Some of the key experimental details are still hard to find in the text. For example, information about how the conditioned medium was collected from the cells (p. 2) could be more explicit, as could the details of filtering the microvesicle prep. At the very least, the information provided in the rebuttal for the reviewers should be included in the Methods section, but it would be even better if the manuscript text and figure legends more clearly explained that the base medium does not contain serum, that the conditioned medium did not contain serum, etc. As is, the figures still seem like the controls and experiments are apples and oranges.*

More experimental detail regarding how conditioned medium was collected and processed, as well as the approach used to isolate microvesicles from embryonic stem (ES) cells (as described in our our previous rebuttal letter) has now been added to Materials and Methods under the subheading *Concentration of CM and MV isolation*. We also more precisely describe in the text (starting with the first paragraph of the Results under the subheading *ES cells generate and shed MVs*) how the ES cell conditioned medium and MVs were generated and then used in biological assays (i.e. the migration assays). Specifically, we explicitly state that to generate conditioned medium, cultures of actively growing ES cells (grown in medium containing serum and leukaemia inhibitory factor (LIF)) were rinsed thoroughly with phosphate buffered saline (PBS), and then placed in medium lacking serum and leukaemia inhibitory factor (LIF); i.e. the *base medium*. Five hours later, this medium was collected off of the cells and centrifuged at low speeds (see Material and Methods for details) to clear the medium of intact cells and large debris. At this point it is referred to as conditioned medium, as it contains only soluble factors and extracellular vesicles secreted by embryonic stem

cells. For all experiments where ES cell conditioned medium was used to treat trophoblasts, the same base medium (again, medium lacking serum and LIF) used to generate the conditioned medium was added to trophoblasts as the control.

Additional information about how microvesicles were isolated, and then used to treat recipient cells, was also included in the revised manuscript (see the fourth paragraph in Results). In it, we highlight how the MVs retained by the 0.22 µm filter were rinsed with PBS and resuspended in serum free medium, and then added to recipient trophoblasts. Thus, in these cases, trophoblasts were treated with the same serum free medium used to to resuspend the MVs as the control.

**Reviewer #3**

*The authors have made a big effort to address all my previous concerns and improved the manuscript significantly. I am satisfied with the revised product.*

**Reviewer #1 (Remarks to the Author)**

The new set of data including trophoblast-MV convincingly strengthens the message and model of the authors.

This article can now be accepted for publication.
